# The true cost of dialysis modalities: A detailed analysis from a UK renal centre

**DOI:** 10.1371/journal.pone.0362592

**Published:** 2026-11-12

**Authors:** Nitin V. Kolhe, Andrew Muggleton, Lisa Page, Richard J. Fluck, Maarten W. Taal

**Affiliations:** 1 University Hospitals of Derby and Burton NHS Trust, Derby, United Kingdom; 2 Centre for Kidney Research and Innovation, Academic Unit for Translational Medical Sciences, School of Medicine, University of Nottingham, Nottingham, United Kingdom; Phramongkutklao College of Medicine, THAILAND

## Abstract

**Purpose:**

Dialysis accounts for more than £1 billion of annual NHS expenditure, yet contemporary modality-specific costing studies using real-world operational data remain limited. Accurate cost estimates are essential for service planning, reimbursement, and workforce allocation. We hypothesised that home-based dialysis modalities, particularly home haemodialysis (HHD) and continuous ambulatory peritoneal dialysis (CAPD), would be less costly and associated with a more favourable cost–benchmark margin than in-centre haemodialysis (ICHD) when workforce, consumables, infrastructure, and overheads were fully allocated.

**Materials and methods:**

A comprehensive micro-costing analysis was undertaken at University Hospitals of Derby and Burton NHS Foundation Trust during 2023–24. Costs were derived from Trust financial ledgers, consultant job plans, workforce activity mapping, consumable expenditure, maintenance, administration, and overhead allocations. Cost-centre and account-code methodologies were used to attribute expenditure to specific dialysis modalities. Costs were compared with NHS Best Practice Tariff reimbursement after adjustment for Market Forces Factor. HHD was evaluated according to treatment frequency, and peritoneal dialysis (PD) costs were analysed by modality.

**Results:**

A total of 45,157 in-centre haemodialysis (ICHD) sessions, 5,326 satellite haemodialysis (SHD) sessions, and 31,737 peritoneal dialysis (PD) treatment days were delivered. For cross-modality comparison, annual per-patient treatment costs were £25,598 for ICHD, £26,678 for SHD, £16,573 for CAPD, £36,859 for CCPD, £41,085 for AAPD, and £18,320, £24,427 and £30,534 for HHD delivered three, four and five times weekly, respectively. CAPD and three-times-weekly HHD had the lowest annual provider costs. Comparisons with NHS Best Practice Tariff (BPT) values were treated as standardised financial benchmarks rather than measures of clinical cost-effectiveness or necessarily as incremental cash reimbursement under the NHS Payment Scheme. Five-times-weekly HHD had a small negative benchmark margin, whereas three- and four-times-weekly HHD had positive margins.

**Conclusions:**

HHD and CAPD were among the lower-cost modalities or were associated with favourable benchmark margins within current English reimbursement structures. This micro-costing methodology provides a reproducible framework for evaluating dialysis costs and informing service planning for home-based dialysis pathways.

## Introduction

Chronic kidney disease affects more than 10% of the UK population and is rapidly becoming more common as the population ages [1,2]. As chronic kidney disease progresses to kidney failure (end-stage kidney disease), dialysis or kidney transplantation is required for survival. The number of people with end-stage kidney disease requiring kidney replacement therapy is increasing worldwide and is predicted to double by 2030 [3]. In 2023 in the UK, there were 31,750 people receiving dialysis [4], 84% on in-centre haemodialysis (ICH), 4.5% on home haemodialysis (HHD) and 11.6% on peritoneal dialysis (PD) [4].

Although therapies like gene therapy have the highest single cost, dialysis is one of the most expensive chronic treatments for the NHS and all healthcare systems because of the large patient population and continuous lifetime treatment requirement. Recent estimates from Kidney Research UK indicate that dialysis represents a major financial commitment for the NHS, costing in excess of £1.05 billion per year, or roughly £34 000 per patient annually in 2023, in addition to £225 million of transport costs for patients receiving dialysis [3]. Given the significant economic burden on health services, it is prudent to determine the true costs of different types of kidney replacement therapies.

There are several approaches to estimating the cost of dialysis. NHS Trusts (hospitals) typically use Patient-Level Information and Costing Systems (PLICS) or aggregated financial data. PLICS applies a bottom-up methodology, assigning costs to individual patients based on actual resource utilisation rather than relying on averages. However, its effectiveness is limited by data quality issues, inconsistent coding, variable clinical engagement, and technical challenges in implementation and integration. As a result, despite improving cost transparency, PLICS data may be constrained by incomplete datasets and a focus on compliance rather than supporting meaningful clinical and operational decision-making.

Another important limitation of micro-costing relates to the accurate measurement of the time spent by medical staff on specific clinical activities. In the UK, NHS consultant’s weekly job plan typically includes a combination of responsibilities such as outpatient clinics, review of dialysis patients, inpatient work, procedures, and administrative or supporting professional activities [5]. When estimating the cost of dialysis services, it is therefore essential to carefully attribute staff time to the relevant dialysis-related tasks. This ensures that workforce costs are allocated appropriately and avoids the risk of overestimating the true cost associated with a particular dialysis activity [6].

This study aimed to undertake a comparative evaluation of the direct expenditure associated with different dialysis modalities, namely automated peritoneal dialysis (APD), continuous ambulatory peritoneal dialysis (CAPD), in-centre haemodialysis (ICHD) delivered within hospitals, haemodialysis provided in satellite units (SHD), and home haemodialysis (HHD). A further objective was to determine the principal factors that influence the overall cost of delivering each modality.

In addition, the analysis sought to map the entire pathway of dialysis care, identifying all activities, resources, and service components involved in managing a patient receiving dialysis to develop a detailed approach to costing that can be applied across the NHS and all healthcare systems.

## Materials and methods

This study was conducted in March 2026 and used detailed financial, workforce, consumable, and activity data from the Renal Department at University Hospitals of Derby and Burton NHS Foundation Trust during the 2023–2024 financial year to undertake a comprehensive micro-costing analysis of all dialysis modalities. University Hospitals of Derby and Burton (UHDB) NHS Foundation Trust provides various renal services across three geographical sites. These activities each have their own cost centre codes**,** which are used to track all the expenses related to a particular service. The workforce, comprised of consultants, resident doctors, nurses, dieticians, maintenance and administrative personnel, provide services across all the above activities. Some consultants and all resident doctors also provide acute medical services and teaching to medical students, funded by Health Education England East Midlands (HEEM). The workforce is grouped according to role and banding, each with an account code**.** Account codes are used to categorize specific financial transactions (income, expenditure, assets, liabilities). These codes can be broken down into different types of costs, such as pay (e.g., staff salaries), non-pay (e.g., drugs, supplies), or overheads (e.g., depreciation).

By combining a cost centre (which shows where a cost occurred) with an account code (which shows what the cost was for), hospitals can gain a detailed view of their financial performance. In the standardised cost ledger, these two elements are mapped together to form a single costing account code, enabling precise analysis and reporting [7,8]. This structured coding supports NHS England’s National Cost Collection, helping to identify the average cost of delivering services and informing payment systems. Overall, this approach ensures transparent financial management and efficient resource allocation to support high-quality patient care.

Workforce costs (consultants, resident doctors, nursing, maintenance and administrative staff) were allocated to specific clinical activities based on job plans, estimated time contribution, and activity mapping across the renal care pathway and is described in detail in the Supplementary material (S1 Text).

We also calculated the cost of all consumables needed for each session of incentre dialysis, including maintenance of the water treatment plant, machine disinfection and any additional consumable for dialysis via tunnelled catheters (permcaths) (S2 Table). We also obtained the cost of non-pay spend for the dialysis unit (S3 Table). We did not include annual maintenance costs for water treatment plant which is maintained by the Private Finance Initiative (PFI) and is included in overheads. We included the annual maintenance cost of the renal electronic patient record and the capitation fees paid to the UK Renal Registry (UKRR) and apportioned that to each dialysis session [6]. We also included the cost of utility bills for patients receiving home therapies [9].

For peritoneal dialysis, we obtained all invoices from the peritoneal dialysis supplier for the consumables and calculated the cost of all types of peritoneal dialysis provided - continuous ambulatory peritoneal dialysis (CAPD), continuous cyclic peritoneal dialysis (CCPD), and assisted automated peritoneal dialysis (AAPD) and cross checked with the account code.

We obtained the total number of haemodialysis sessions by access type (fistula or permcath) and blood-borne virus (BBV) status to account for isolation requirements, recognising that dialysis for patients with infections (e.g., HIV, hepatitis B or C) incurs higher costs due to enhanced infection control measures and increased clinical management needs. Similarly, we obtained the number of days on different types of peritoneal dialysis to calculate the cost per day

Local institutional overhead rates supplied by UHDB Finance were applied to account for indirect organisational and infrastructure costs not directly attributable to an individual dialysis treatment. A 30% uplift was applied to haemodialysis delivered at Royal Derby Hospital and a 15% uplift to community, satellite and home therapies. These are local UHDB costing assumptions rather than nationally mandated NHS overhead rates. The overheads are intended to represent residual shared organisational costs, such as corporate support and infrastructure, after direct modality-specific costs have been identified. Directly measured staffing, consumables, equipment, utilities, maintenance and administrative costs were reviewed separately to minimise double counting.

We compared estimated provider costs with the 2023–24 NHS Best Practice Tariff (BPT) values, adjusted for the Trust Market Forces Factor (MFF). Accordingly, BPT values in this study were used as a standardised benchmark for comparing modality-specific provider costs and should not be interpreted as the actual incremental cash reimbursement received by the Trust for each treatment. We therefore describe differences between estimated cost and BPT as ‘benchmark margins’ rather than accounting surpluses or deficits.

### Sensitivity analysis

#### Overhead sensitivity analysis.

The precise composition of the local percentage uplifts cannot be disaggregated retrospectively from the institutional rate. To test the influence of the differential institutional overhead assumptions, we reconstructed pre-overhead annual costs from the base-case estimates and applied common overhead rates of 0%, 15% and 30% to all modalities (S4A Table). This scenario analysis was designed to test whether the relative cost ranking was driven by the local 30%/15% differential rather than underlying modality costs.

#### HHD sensitivity analysis.

A sensitivity analysis was undertaken to assess the effect of treatment frequency on HHD costs under an alternative cost-allocation assumption. The primary analysis cost for HHD delivered three times weekly was retained as the baseline. All non-equipment programme costs were assumed to remain unchanged with increasing dialysis frequency, while equipment costs were assumed to increase with each additional dialysis session (S4B Table). The incremental equipment cost per treatment was calculated from annual HHD equipment expenditure (£1,090,869.91) divided by the total number of HHD sessions (13,520). The same 15% overhead used in the primary HHD analysis was applied to the incremental equipment cost. This analysis represented an alternative modelling scenario and did not replace the primary HHD costing methodology.

## Results

### In-centre haemodialysis

During 2023–24, a total of 45,157 haemodialysis sessions were delivered across the renal service. The total annual expenditure was £5.70 million, comprising £3.39 million (60%) in nursing pay, £1.92 million (33%) in consumables and equipment, and the remainder in medical staff cost (5%), maintenance (1%), and administrative support (1%). Although dialysis consumable costs could be differentiated by vascular access type, granular costs for other non-dialysis consumables, nursing, and the medical workforce could not be reliably apportioned across individual access categories. Therefore, average staffing and operational costs were applied to all dialysis sessions, regardless of access type. Following full cost allocation, including a 30% overhead uplift, the final mean cost per session was £164.09 across all access categories (Table 1).

**Table 1:** In-centre haemodialysis: cost per session and margin (2023–24).

Access type*	Sessions	Final cost (£)	BPT^†^ with MFF^‡^ (£)	Benchmark margin (BPT – cost)
AV fistula	36,151	164.09	193.44	£29.35
AV Fistula with BBV infection	336	164.09	217.36	£53.27
Central venous catheter	8,436	164.09	144.56	-£19.53
Central venous catheter + BBV ^§^	234	164.09	172.64	£8.55

§BBV=blood borne virus; ^†^BPT=Best Practice Tariff; ^‡^MFF= Market Forces Factor.

*Small differences in consumable costs were averaged across incentre HD with different access options.

Haemodialysis consumable costs varied according to dialysis delivery approach and vascular access type, including double-needle, single-needle, blood-borne virus (BBV)-positive, and central venous catheter (CVC)-based haemodialysis. The weighted mean haemodialysis consumable cost across all haemodialysis sessions was £42.53 per session (Table 2).

**Table 2:** Cost of consumables for haemodialysis.

Haemodialysis (HD) access and approach	Consumable cost per dialysis session
HD – Double Needle	£39.07
HD – Single Needle	£50.80
HD with BBV Infection	£36.36
HD - central venous catheter (CVC)	£37.04
Average	£42.53

Relative to the BPT benchmark, AVF and CVC categories showed different benchmark margins because the reimbursement values differ by access type, while the same mean total delivery cost (£164.09/session) was applied across access categories. The analysis therefore does not demonstrate an access-specific difference in total provider cost. Although access-specific consumable costs were measured, the CVC consumable estimate (£37.04/session) was below the overall mean (£42.53/session); consequently, the negative CVC benchmark margin should not be attributed to higher consumable costs. Access-specific margin findings are presented as reimbursement benchmarking only.

### Satellite haemodialysis unit

At the satellite haemodialysis unit, 5,326 sessions were performed (443 sessions via CVC) at a total cost of £910,822.14, equating to £171.01 per session after a 15% overhead allocation. We were unable to separate costs for haemodialysis performed via AVF or CVC and therefore averaged the BPT for AVF and CVC. The corresponding BPT benchmark values for dialysis sessions via AVF and CVC were £944,567.50 and £64,040.08, respectively, corresponding to a positive aggregate benchmark difference of £97,785.50

### Peritoneal dialysis

The total annual activity for peritoneal dialysis (PD) was 31,737 treatment days. Using a 15% overhead adjustment, continuous ambulatory PD (CAPD) had the lowest cost per day, while machine-based therapies were more resource-intensive (Table 3).

**Table 3:** Peritoneal dialysis: cost and reimbursement (2023–24).

Modality	Treatment days	Cost/day (£)	BPT/day (£)	Benchmark margin/day (£)	Benchmark margin (£)
CAPD	23,421	45.53	56.16	+10.63	248,998.25
CCPD	5,209	101.26	67.60	−33.66	−175,319.25
AAPD	3,107	112.87	107.12	−5.75	−17,859.22
Total PD	31,737	—	—	—	+55,819.78

AAPD = assisted automated peritoneal dialysis; CAPD = continuous ambulatory peritoneal dialysis; CCPD = continuous cyclic peritoneal dialysis.

CAPD had the lowest observed provider cost among PD modalities (£45.53/day). CCPD (£101.26/day) and AAPD (£112.87/day) had higher observed treatment costs and negative benchmark margins. These are descriptive cost differences; no statistical comparison between modalities was performed. The larger CCPD and AAPD costs reflect the actual modality-specific supplier expenditure and additional machine-based therapy costs captured in the Trust accounts, including cycler/equipment-related and delivery costs. Contract structure and equipment pricing are centre-specific and may limit generalisability.

### Home haemodialysis

During 2023–24, the number of patients receiving home haemodialysis (HHD) fluctuated between 57 and 62, with 62 patients receiving treatment during the majority of weeks. Accordingly, 62 patients were used for the activity-based costing analysis, comprising 15 patients dialysing three times weekly, 20 four times weekly, and 27 five times weekly. This corresponds to 13,520 annual sessions under the frequency distribution used in the model. The direct annual programme cost was £1,380,652.03. After applying the 15% institutional overhead, the total annual HHD programme cost was £1,587,749.83, corresponding to a frequency-weighted mean cost of £492.48 per patient-week (Table 4). The primary activity-based analysis estimated weekly costs of £352.31, £469.75 and £587.19 for HHD delivered three, four and five times weekly, respectively. HHD equipment and programme costs were allocated across delivered/modelled activity; the analysis did not assume that higher treatment frequency diluted fixed costs.

**Table 4:** Cost of home HD as per number of sessions per week.

Group	Patients	Sessions/week	Weekly Cost per Patient	BPT	Benchmark margin/week
Group 1	15	3	£352.31	£573.24	£220.93
Group 2	20	4	£469.75	£573.24	£103.49
Group 3	27	5	£587.19	£573.24	-£13.95
Total	62	–	£492.48	£573.24	£80.76

Using the BPT as a financial benchmark, HHD delivered three or four times weekly had positive benchmark margins, whereas five-times-weekly HHD had a small negative benchmark margin. Increasing treatment frequency increased the modelled weekly provider cost because the costing approach allocated programme expenditure across sessions; therefore, no inference is made that higher frequency produced fixed-cost dilution or improved economic efficiency

### HHD sensitivity analysis

The calculated equipment cost was £80.69 per additional dialysis session before overhead and £92.79 after application of the 15% overhead. Using the primary three-times-weekly HHD cost of £352.31 per patient-week as the baseline, estimated weekly costs were £352.31, £445.10 and £537.89 for HHD delivered three, four and five times weekly, respectively. Corresponding costs per dialysis session were £117.44, £111.27 and £107.58, respectively (S4B table). Thus, although total weekly cost increased with dialysis frequency, the average cost per session decreased under this alternative allocation assumption because non-equipment programme costs were held constant while equipment costs increased with additional treatments. (S4B Table).

### Overall findings

Table 5 highlights variation in annual provider costs across dialysis modalities. CAPD was the least expensive modality (£16,573/patient-year), followed by HHD delivered three times weekly (approximately £18,320/patient-year). HHD four times weekly cost approximately £24,427/year, ICHD £25,598/year, SHD £26,678/year, and HHD five times weekly approximately £30,534/year. CCPD (£36,859/year) and AAPD (£41,085/year) had the highest observed annual costs. Benchmark margins were positive for CAPD, three- and four-times-weekly HHD, and selected HD BPT categories; five-times-weekly HHD had a small negative benchmark margin. As HHD receives a fixed weekly BPT irrespective of treatment frequency, its benchmark margin decreased with increasing dialysis frequency. Because the same mean delivery cost was applied to AVF and CVC categories within each of the ICHD and SHD settings, access-specific differences in benchmark margin reflect differences in BPT values rather than independently estimated access-specific total costs.

**Table 5:** Comparative costs and benchmark margins across dialysis modalities. All costs in GBP (£). Weekly benchmark margin = weekly BPT − estimated weekly cost*.

Dialysis modality	Estimated unit cost	BPT (payment unit)	Weekly cost	Weekly BPT	Weekly margin	Annual cost	Benchmark margin
ICHD via AVF	£164.09/session	£193.44/session	£492.27	£580.32	+£88.05	£25,598.04	Positive
ICHD via AVF + BBV	£164.09/session	£217.36/session	£492.27	£652.08	+£159.81	£25,598.04	Positive
ICHD via CVC	£164.09/session	£144.56/session	£492.27	£433.68	−£58.59	£25,598.04	Negative
ICHD via CVC + BBV	£164.09/session	£172.64/session	£492.27	£517.92	+£25.65	£25,598.04	Positive
SHD via AVF	£171.01/session	£193.44/session	£513.03	£580.32	+£67.29	£26,677.56	Positive
SHD via CVC	£171.01/session	£144.56/session	£513.03	£433.68	−£79.35	£26,677.56	Negative
CAPD	£45.53/day	£56.16/day	£318.71	£393.12	+£74.41	£16,572.92	Positive
CCPD	£101.26/day	£67.60/day	£708.82	£473.20	−£235.62	£36,858.64	Negative
AAPD	£112.87/day	£107.12/day	£790.09	£749.84	−£40.25	£41,084.68	Negative
HHD 3/week	£117.44/session	£573.24/week	£352.31	£573.24	+£220.93	£18,320.12	Positive
HHD 4/week	£117.44/session	£573.24/week	£469.75	£573.24	+£103.49	£24,427.00	Positive
HHD 5/week	£117.44/session	£573.24/week	£587.19	£573.24	−£13.95	£30,533.88	Negative

Abbreviations: AAPD, assisted automated peritoneal dialysis; AVF, arteriovenous fistula; BBV, blood-borne virus; BPT, Best Practice Tariff; CAPD, continuous ambulatory peritoneal dialysis; CCPD, continuous cyclic peritoneal dialysis; CVC, central venous catheter; HHD, home haemodialysis; ICHD, in-centre haemodialysis; SHD, satellite haemodialysis.

*Estimated treatment costs and BPT values are expressed according to the relevant modality-specific units. For in-centre and satellite haemodialysis, both costs and BPT values are reported per dialysis session, whereas for peritoneal dialysis, they are reported per treatment day. For home haemodialysis (HHD), estimated costs are expressed per session, while the BPT is a fixed weekly payment irrespective of dialysis frequency. Weekly costs were calculated assuming three sessions for in-centre and satellite haemodialysis, three, four or five sessions for HHD, and seven treatment days for peritoneal dialysis. Annual costs were calculated using 52 weeks of treatment. The weekly benchmark margin represents the difference between the weekly BPT and estimated weekly provider cost. A positive margin indicates costs below the BPT benchmark, while a negative margin indicates costs exceeding the benchmark. BPT values were used as standardised financial comparators and do not necessarily represent actual reimbursement received by the Trust. Estimated modality costs are based on average resource utilisation and may not capture all patient-specific variations.

### Sensitivity analysis

When common overhead rates of 0%, 15% and 30% were applied across all modalities, absolute annual costs changed as expected but the broad ranking was stable. CAPD and three-times-weekly HHD remained the two lowest-cost modalities in every scenario, while CCPD and AAPD remained the two highest-cost modalities. Thus, the principal ranking was not dependent on the differential 30% hospital/15% community-home overhead assumption (S4A Table).

## Discussion

This single-centre provider-perspective micro-costing analysis demonstrates substantial variation in observed costs and BPT benchmark margins between dialysis modalities. CAPD and three times weekly HHD had the lowest annual provider costs in this service, whereas CCPD and AAPD had the highest. These findings describe costs within the local UHDB staffing, procurement, estate and overhead structure and do not constitute a formal cost-effectiveness analysis.

Economic modelling has also examined the system-level value of dialysis modalities. A Value in Health analysis of an integrated home-dialysis strategy in the UK suggested improved lifetime cost-effectiveness by increasing HHD uptake among suitable patients, reinforcing the budget impact of shifting activity away from in-centre care [10]. Outside the UK, contemporary comparative studies similarly report lower weekly global costs for daily HHD than for in-centre HD, aligning with the UK pattern of home-based cost advantages [11]. Broader reviews of CKD economics further show that dialysis represents a sharp step-up in per-patient cost compared with earlier CKD stages, not only underscoring the importance of kidney protection in early CKD stages, but also, modality choice when the need for RRT arises [12].

At the macro level, published UK estimates indicate that dialysis and transport for in-centre haemodialysis impose substantial health-system expenditure. Transport was not included in our Trust-level micro-costing model; therefore, any reduction in transport expenditure associated with home therapies represents a potential additional system-level benefit rather than an observed saving in the present analysis. Published transport estimates are discussed only to place the provider-perspective findings in wider context.

Recently, Kidney Research UK estimates that dialysis accounted for ~£1.05 billion of NHS spending in 2023 (≈£34,000 per patient-year), with transport for in-centre dialysis alone contributing ~ £225 million annually—a system cost largely avoided by home therapies [3]. NHS England’s universal transport support policy for in-centre HD patients further illustrates the scale and centrality of transport in the overall cost footprint [13]. Depending on geography, the average cost of transport for in-centre dialysis patients is estimated to range from £25–£35 per return journey, equating to £75–£105 per patient per week (based on three sessions weekly) [14]. Taken together, these studies consistently indicate that PD—particularly CAPD—and HHD tend to be less costly to deliver than in-centre HD, with the cost gap widening when transport and other pathway-specific overheads are included*.*

The HHD sensitivity analysis also demonstrated the importance of cost-allocation assumptions. When non-equipment programme costs were assumed to remain unchanged per patient-week and equipment costs were assumed to vary with treatment frequency, weekly costs increased with dialysis frequency while estimated cost per session decreased as the unchanged non-equipment programme costs were distributed across more treatments. This alternative scenario illustrates how HHD cost estimates by treatment frequency are influenced by the underlying cost-allocation assumptions.

An earlier Welsh study from 2008 reported annual costs of £35,023 for hospital haemodialysis and £32,669 for satellite haemodialysis, compared with £21,655 for automated peritoneal dialysis (APD), £15,570 for continuous ambulatory peritoneal dialysis (CAPD), and £20,764 for home haemodialysis (HHD), demonstrating lower costs for home-based modalities [15]. This study used a mixed costing methodology combining micro-costing and top-down approaches, with clinical pathways informed by semi-structured interviews and supplemented by published cost sources where local data were unavailable. A more recent Welsh analysis from 2022 reported lower absolute costs, including approximately £16,395 for CAPD, £20,295 for APD, and £23,403 for HHD, while in-centre haemodialysis costs increased substantially when transport costs were included [16].

Compared with these studies, our methodology used substantially more granular workforce attribution based on consultant job plans, programmed activities, trainee allocation, administrative workload, kidney care team activity, and organisational pay and non-pay expenditure mapping. Unlike prior studies that relied on interview-derived pathways, workforce surveys, or national costing datasets, our study directly apportioned workforce and operational costs using real-world service delivery data within a large NHS renal centre. In addition, we included the cost of utilities and capitation fee to UK renal registry [6,9]. Despite methodological differences, these studies generally support the finding that selected home-based dialysis modalities, particularly CAPD and HHD, can be less costly than in-centre haemodialysis.

Dialysis reimbursement in the NHS has historically sat within the wider national payment architecture, under which activity was grouped into clinically similar currencies and priced using nationally determined rules derived from cost data submitted by providers [17]. Healthcare Resource Groups (HRGs) classify episodes of care into clinically similar categories with comparable resource use, derived from routinely recorded diagnostic (ICD-10) and procedural (OPCS-4) codes. These groupings act as standardised units of healthcare activity and have been used across the NHS to support consistent commissioning and reimbursement. Under the previous tariff framework, HRG-based currencies underpinned payment for many hospital services by linking reimbursement to activity and case mix; however, the NHS payment architecture has since transitioned from the National Tariff Payment System to the NHS Payment Scheme from 1 April 2023 [17,18]. The aligned payment and incentive (API) arrangement combines fixed and variable elements rather than relying solely on a purely activity-based model. NHS England states that API is a form of blended payment designed to support financial stability while still retaining incentives linked to activity and service delivery [19,20].

For dialysis services, this policy shift is important because renal care is a high-volume, long-term, resource-intensive pathway, and reimbursement mechanisms based only on average national prices may not fully capture the real cost of delivering treatment in different organisations. Local cost pressures may vary according to staffing models, transport arrangements, consumable prices, estate costs, utility use, satellite provision, and the relative balance between in-centre, satellite, peritoneal, and home-based dialysis services. The NHS Payment Scheme recognises that currencies group together activities with broadly similar resource needs and costs, but such groupings inevitably simplify real-world variation in service delivery.

Taken together, these changes mean that the costing of dialysis should not be interpreted only through the lens of tariff or contract value. A robust assessment of dialysis costs requires detailed understanding of the treatment pathway, modality-specific resource use, and patient-level or service-level cost variation, particularly when comparing home haemodialysis, hospital haemodialysis, satellite haemodialysis, and peritoneal dialysis models.

### Strengths and limitations

#### Strengths.

This costing exercise was undertaken in greater detail than most published analyses, incorporating all pay and non-pay components with explicit overhead application. The model also includes reimbursement for domestic utilities (e.g., water, electricity) which reflects the true home-therapy costs [9].

#### Limitations.

As a single-centre analysis, findings may not generalise to institutions with different staffing or procurement structures. We used average cost of medical, nursing and equipment for haemodialysis with various access and BBV and were unable to get granular costs of haemodialysis with different type of vascular access and those infected with blood borne virus. Additionally, patient transport and system-level commissioner reimbursements were not included in the Trust-level analysis. In addition, patient populations receiving in-centre and home dialysis may differ in age, comorbidity and clinical complexity. As this was a service-level costing study, we did not adjust costs for differences in patient-level case mix between dialysis modalities. The HHD sensitivity analysis assumed that non-equipment programme costs remained unchanged across treatment frequencies and that equipment costs varied with the number of dialysis sessions; actual cost behaviour may be more complex, and the analysis should therefore be interpreted as an alternative costing scenario rather than an observed frequency-specific cost structure.

### Implications for policy and practice

Within this Trust-level analysis, CAPD and lower-frequency HHD were associated with comparatively low provider costs. Potential benefits of home therapies for transport expenditure, estate use, autonomy or quality of life were not measured in this study and should be evaluated separately when considering wider service redesign.

## Conclusion

This detailed provider-perspective micro-costing analysis found that CAPD and lower-frequency HHD had comparatively low annual delivery costs within UHDB, while CCPD and AAPD had the highest observed annual costs. Comparisons with BPT values are presented as standardised benchmark margins rather than formal measures of cost-effectiveness or actual incremental reimbursement. The results should be interpreted as a detailed real-world example from one NHS Trust. The costing framework could be adapted and externally validated in other centres using local staffing, procurement, estate, programme-size and overhead assumptions.

**Key learning points**

**What was known**

- Dialysis costs the NHS over £1 billion annually, yet contemporary UK studies providing detailed modality-specific costing remain limited. Home-based therapies have generally been reported to be less costly than in-centre haemodialysis.
- Previous UK cost analyses used mixed micro-costing and top-down methodologies, often relying on interviews, workforce surveys, or national datasets rather than detailed operational service data.
- Workforce costs are a major component of dialysis expenditure, but accurately attributing staff time across dialysis, inpatient care, teaching, administration, and governance activities remains challenging.

**This study adds**

- This study used detailed job-plan analysis, workforce mapping, financial ledger data, and activity-based costing to provide a comprehensive real-world assessment of dialysis costs within a large NHS renal centre.
- Home haemodialysis (HHD) and continuous ambulatory peritoneal dialysis (CAPD) were associated with comparatively low provider costs or favourable benchmark margins under current English reimbursement arrangements.
- Different vascular-access categories were associated with different BPT benchmark margins; however, access-specific total provider costs could not be estimated.

**Potential impact**

- Expanding home dialysis programmes may reduce provider resource use and could offer additional system-level benefits, including reduced transport requirements; these wider effects were not measured in this study.
- Vascular access categories were associated with different BPT benchmark margins, although access-specific total provider costs were not estimated.
- The costing methodology provides a reproducible framework to support service redesign, tariff evaluation, workforce planning, and dialysis policy development across healthcare systems.

## Supporting information

S1 Text Workforce costing for dialysis. (DOCX)

S2 Table Consumables needed for each session of in-centre dialysis. (DOCX)

S3 Table Non-pay spend for the dialysis unit. (DOCX)

S4A Table Sensitivity analyses of alternative overhead assumptions. (DOCX)

S4B Table HHD sensitivity analysis assuming unchanged non-equipment costs and frequency-dependent equipment costs. (DOCX)

## References

[1] Health Survey for England. Available from: https://digital.nhs.uk/data-and-information/publications/statistical/health-survey-for-england/health-survey-for-england-2016

[2] Hirst JA, Hill N, O’Callaghan CA, Lasserson D, McManus RJ, Ogburn E, et al. Prevalence of chronic kidney disease in the community using data from OxRen: A UK population-based cohort study. Br J Gen Pract. 2020;70(693):e285–93. doi: 10.3399/bjgp20X708245 32041766 PMC7015167

[3] KRUK. Available from: https://www.kidneyresearchuk.org/wp-content/uploads/2023/06/Economics-of-Kidney-Disease-full-report_accessible.pdf

[4] UKRR. Available from: https://www.ukkidney.org/sites/default/files/documents/Adult%20Data.pdf

[5] BMA. Available from: https://www.bma.org.uk/pay-and-contracts/job-planning/job-planning-process/an-overview-of-job-planning?utm_source=google&utm_medium=search&utm_campaign=consultant_charter_2026&utm_content=job_planning&campaigncode=5461&gad_source=1&gad_campaignid=23676173236&gclid=Cj0KCQjw1ZjOBhCmARIsADDuFTDosIjLfIgnhG1Ox7fizUy2-Gksx-OLq7ailNt_4HudBx4W1E2cD38aAvtiEALw_wcB

[6] NHS england. Renal dialysis - costing extension. Available from: https://www.england.nhs.uk/wp-content/uploads/2021/03/Renal-Dialysis-CA3-2021.pdf

[7] NHS England. Available from: https://www.england.nhs.uk/wp-content/uploads/2020/08/Quick_start_guide_for_the_healthcare_costing_standards_2018.pdf

[8] HFMA. Available from: https://www.hfma.org.uk/system/files?file=understanding-the-general-ledger-for-costing---guidance-paper-february-2016.pdf&sfvrsn=0

[9] UKKA. Available from: https://www.ukkidney.org/about-us/news/reimbursement-additional-utility-costs-patients-receiving-home-dialysis

[10] Erbe AW, Kendzia D, Busink E, Carroll S, Aas E. Value of an integrated home dialysis model in the United Kingdom: A cost-effectiveness analysis. Value Health. 2023;26(7):984–94. doi: 10.1016/j.jval.2023.02.009 36842716

[11] Rostoker G, Dumas J-J, Thomé B, Aragno V-A, Thomas M, Canaud B. Cost-effectiveness of daily home haemodialysis versus in-centre haemodialysis: A propensity score matching analysis based on real-world data from the French healthcare system. Eur J Intern Med. 2025;137:73–82. doi: 10.1016/j.ejim.2025.04.017 40318912

[12] Jha V, Al-Ghamdi SMG, Li G, Wu M-S, Stafylas P, Retat L, et al. Global economic burden associated with chronic kidney disease: a pragmatic review of medical costs for the inside CKD research programme. Adv Ther. 2023;40(10):4405–20. doi: 10.1007/s12325-023-02608-9 37493856 PMC10499937

[13] NHS England. https://www.england.nhs.uk/commissioning/spec-services/npc-crg/group-a/renal-services/transport-support-for-patients-attending-in-centre-haemodialysis/?utm_source=chatgpt.com

[14] NHS England. https://www.england.nhs.uk/wp-content/uploads/2021/08/B0682-fnal-report-of-the-non-emergency-patient-transport-review.pdf

[15] Baboolal K, McEwan P, Sondhi S, Spiewanowski P, Wechowski J, Wilson K. The cost of renal dialysis in a UK setting--a multicentre study. Nephrol Dial Transplant. 2008;23(6):1982–9. doi: 10.1093/ndt/gfm870 18174268

[16] Roberts G, Holmes J, Williams G, Chess J, Hartfiel N, Charles JM, et al. Current costs of dialysis modalities: A comprehensive analysis within the United Kingdom. Perit Dial Int. 2022;42(6):578–84. doi: 10.1177/08968608211061126 35068280

[17] NHS England. Past National Tariffs: Documents and Policies. Available from: https://www.england.nhs.uk/publication/past-national-tariffs-documents-and-policies

[18] NHS England. 2020-21 National Pay Tariff System. Available from: https://www.england.nhs.uk/wp-content/uploads/2021/02/20-21_National-Tariff-Payment-System.pdf

[19] NHS England. 2025/26 NHS Payment Scheme – NHS provider payment mechanisms. Available from: https://www.england.nhs.uk/long-read/25-26-nhsps-annex-b-guidance-on-currencies

[20] NHS England. 2025/26 NHS Payment Scheme – a consultation notice. Available from: https://www.england.nhs.uk/long-read/2025-26-nhs-payment-scheme-consultation

