# Supplementary material for: The true cost of dialysis modalities: A detailed analysis from a UK renal centre
Source: PLoS One. 2026 Nov 12;21(11):e0362592. doi: 10.1371/journal.pone.0362592 (PMC13759095; doi:10.1371/journal.pone.0362592)
Supplement: Supplementary file 1 [file pone.0362592.s001.docx]

**S1 Text: Workforce Costing for dialysis**

The detail on the workforce costing methodology underpinning the estimates presented in the main manuscript is provided below. This section expands on the principles already described, outlining how workforce contributions were apportioned across renal activities; however, it does not alter the overall costing framework or results. The detailed methodology describes the allocation of consultant, resident doctor, nursing, kidney care team, dietetic, maintenance, and administrative inputs across multiple clinical and supporting activities, reflecting the complexity of shared roles and cross-cover within renal services. Workforce members often contribute across several services, including acute medical care, renal services at peripheral sites such as Queen’s Hospital Burton, and undergraduate teaching funded by Health Education England East Midlands (HEEM). All staff groups are categorised according to role and banding and mapped to corresponding account codes to enable consistent and transparent attribution of costs across activities.

**Calculation of cost for renal activities**

In UHDB, the contribution of the renal medicine workforce to each activity can vary depending on their job plans (for consultants), acute medical commitments (resident doctors) and working across multiple activities with varying time commitment (kidney care team). Resident doctors are funded by Health Education East Midlands (HEEM) and the Hospital for out-of-hours work, and they support various services with majority of their work on inpatient wards followed by incentre haemodialysis and peritoneal dialysis. Resident doctors do not have time commitment to home haemodialysis. Undergraduate teaching and acute medicine commitments are paid separately, while administrative commitment is divided across different tasks such as letter typing, calendar management, and answering patient phone calls.

We reviewed the job plans for all consultants and calculated the number of hours, across various areas including inpatient care, teaching, acute medicine, satellite units, the renal assessment unit, procedures, work in peripheral sites without dialysis units, and transplant activities. Many activities are provided by more than 1 consultant. The activities provided by consultants are as follows:

- For each renal replacement therapy (RRT) modality - In-centre haemodialysis (ICHD), home haemodialysis (HHD), peritoneal dialysis (PD) and transplantation, Consultants lead monthly multidisciplinary team (MDT) patient reviews, answer queries related to RRT modality and perform RRT modality outpatient clinics.
- For satellite dialysis units- Satellite HD clinic and MDT patient reviews
- For sites without a dialysis unit – inpatient referrals and clinics.
- Procedures - vascular catheter and PD catheter placement, and kidney biopsies
- Teaching undergraduate medical students
- Renal assessment unit – patient assessment
- Inpatient cover for renal and medical wards
- General Nephrology and low clearance outpatient clinics
- Acting as clinical lead for clinical governance activities

Consultant costs for all clinical activities were categorised and calculated from programmed activity (PA) as follows:

- Costing for QHB nephrology and SJH (nephrology and RRT) clinics were calculated separately. ICHD, PD, HHD, LCC and transplant clinics were treated independently for modality costing from their respective modalities, as they are remunerated separately. Nephrology clinics were grouped together for costing as they have different renumeration from other RRT clinics.
- Respective MDTs were grouped together with activities for low clearance clinic (LCC), ICHD, PD, HHD, transplant clinics as the MDTs are not funded but they incur a cost.
- Procedures have distinct remuneration arrangements, and workforce contributions; the costs for these were calculated separately.
- Service increment for teaching (SIFT) PAs for teaching medical students was separated as it is funded by HEEM.
- Cost for proving inpatient activities on renal, medical wards and renal assessment units was calculated from allocated PAs and were grouped together.
- PAs for being a lead clinician for certain activities were grouped with that particular activity and for activity independent lead was distributed across all activities being performed by consultants.
- During inpatient sessions, other routine clinical activities were cancelled, and adjustments were made for any sessions cancelled due to inpatient commitments in line with the inpatient work timetable
- The PAs for each activity mentioned above were added and the total of all consultant's PAs for administrative and supporting professional activities (SPA) were proportioned to these activities to get final total PAs needed for each activity
- We calculated the proportion of PA needed for each activity from the grand total PA for all renal consultants.
- We calculated the consultant cost for each activity from the total pay and non-pay spend
- We excluded Consultant oncall Pas as majority of the oncall activity is related to medical high dependency unit.

Resident doctors mainly work on the wards, with most of their costs reimbursed by HEEM. However, some activity-related costs are not reimbursed. It was assumed that 10% of their time is dedicated to acute medicine, based on an on-call rota of approximately 1:10. Their contribution to peritoneal dialysis (PD) is minimal, at around 5%, and they do not contribute to home haemodialysis (Home HD). Over the year, they conducted 171 clinics, representing about 1.5% of their activity, while their contribution to haemodialysis (HD) is estimated at 14%. The majority of their work, approximately 70%, is focused on inpatient care. Based on above proportions, the cost of resident doctors was calculated for each activity.

The administrative team did not contribute directly to any clinical activity but are important members of the renal specialty allowing the smooth running of the department. We calculated the number of letters typed for HD, PD, HHD, and Transplant (excluding QHB and SJH), and the associated cost was allocated based on the number of letters produced, using a rate of 12 letters per hour. The administrative staff dedicate one whole-time equivalent (WTE) each day to handling telephone calls, with the remainder of their time spent managing diaries. The time and cost for these activities are distributed equally across HD, Transplant, PD, HHD, and Nephrology. While some variation is expected, it is not anticipated to have a significant impact on overall costs.

The three nurses in the Kidney Care Team (KCT) provide services for PD, HHD, Clinics, dialysis unit, anaemia clinic and inpatient wards in varying proportion. These KCT costs were calculated from the total pay and non-pay spend by using the proportion of time spent for each activity.

Cost of Nurses proving transplant and outpatient services were added to respective activities, while costs pertaining to AKI nurse was separated.

**Overhead allocation, HHD programme costing and sensitivity analysis**

Overhead allocation. The 30% hospital and 15% community/satellite/home overhead uplifts are local institutional rates supplied by UHDB Finance and are not national NHS-mandated percentages. They are intended to capture residual indirect organisational and infrastructure costs that cannot be directly attributed to an individual dialysis treatment. Directly identified modality-specific costs—including clinical workforce, consumables, equipment, utilities, maintenance and administration where separately measured—were costed before application of the institutional uplift. The costing model was reviewed to minimise double counting. Because the institutional percentage rates could not be retrospectively disaggregated into individual corporate cost components, their effect was tested by sensitivity analysis.

**Capital, equipment and programme costs.** We used the actual costs recorded by UHDB during the 2023–24 financial year. Costs for dialysis equipment and service contracts were taken directly from the Trust financial records and supplier invoices. Where these were already recorded as annual costs, no further adjustment for equipment lifespan or depreciation was made. Training and programme set-up costs were included only if they were incurred and recorded during 2023–24. We did not estimate additional costs for unused dialysis capacity. Therefore, the costs reported in this study represent the actual expenditure incurred in delivering the dialysis programme during 2023–24. Costs may differ in other centres or where the number of patients, equipment contracts or service arrangements are different. HHD allocation. During 2023–24, the number of patients receiving HHD varied between 57 and 62 across the year with 62 patients receiving treatment during the majority of weeks. The treatment-frequency distribution used for the activity model comprised 15 patients at three sessions/week, 20 at four sessions/week and 27 at five sessions/week, corresponding to 13,520 modelled annual sessions and 3,224 patient-weeks. The direct annual programme cost was £1,380,652.03 and, after application of the 15% institutional overhead, the total annual programme cost was £1,587,749.83. This corresponds to a frequency-weighted mean cost of £492.48 per patient-week. The primary activity-based analysis estimated weekly costs of £352.31, £469.75 and £587.19 for HHD delivered three, four and five times weekly, respectively.

Peritoneal dialysis. PD consumable and equipment expenditure was derived from actual supplier invoices and cross-checked against the relevant Trust account codes. The higher observed costs of CCPD and AAPD compared with CAPD therefore reflect the modality-specific expenditure recorded under the local supplier arrangements, including machine-based therapy costs. These contract-specific differences may not generalise to centres with different procurement or equipment arrangements.
