# Supplementary material for: The true cost of dialysis modalities: A detailed analysis from a UK renal centre
Source: PLoS One. 2026 Nov 12;21(11):e0362592. doi: 10.1371/journal.pone.0362592 (PMC13759095; doi:10.1371/journal.pone.0362592)
Supplement: Supplementary file 2 [file pone.0362592.s002.docx]

**S2 table: Consumables needed for each session of incentre dialysis**

| **Equipment - 1** | **Equipment - 2** | **Central Venous Catheter** | **Additional equip** | **Disinfection** |
| --- | --- | --- | --- | --- |
| Double needle | Chlorhexidine 200ml | 10ml syringe | Re-circulator | C-Cart |
| Single needle | Dialysate | Combi-lock same as cap | Infusion lines | A-Cart |
| Dialyser | Bicart | Dressing (Tegaderm with Chlorhexidine gel) | Blood transfusion lines | Acticlor tablets |
| Normal saline 1 Litre | Arlaid/Sani clean wipes | Dressing (Clear film dressing without Chlorhexidine gel) | Oxygen mask | Detergent Soap (L) |
| Normal saline 500ml | Gloves | Chlora Prep | Yankauer Suction |  |
| 5 ml syringe | Apron | Chlorhexidine Alcohol swab | Tubing set for Yankauer Suction Liner |  |
| Blunt needle | Tape | TPE 2000 set |  |  |
| Blunt needle with filter | Sharp box |  |  |  |
| Fistula needle - blunt OR | QA lines |  |  |  |
| Fistula needle - sharp | Caps |  |  |  |
| Put on pack | Mupirocin per tube |  |  |  |
| Take off pack | Y Connector (Single needle HD) |  |  |  |
| Enoxaparin | HD machine |  |  |  |
|  | Sterile Scissors |  |  |  |
