# Supplementary material for: The true cost of dialysis modalities: A detailed analysis from a UK renal centre
Source: PLoS One. 2026 Nov 12;21(11):e0362592. doi: 10.1371/journal.pone.0362592 (PMC13759095; doi:10.1371/journal.pone.0362592)
Supplement: Supplementary file 3 [file pone.0362592.s003.docx]

**S3 table: Non-pay spend for the dialysis unit**

| Med & Surg Equipment - Purchase | Provisions & Beverages | Cleaning Items & Toiletries | Taxis - Staff & Equipment |
| --- | --- | --- | --- |
| Med & Surg Consumables | Clothing & Uniforms | Electricity | Taxis - Specimens & Drugs |
| Dressings | Delivery Charges | Furnishings & Fittings | Training |
| All Other Appliances | Printing & Stationery | Office Equipment | Patients Taxis |
| Med & Surg Equipment - Repairs | Photocopies | Computer Hardware & Software - Purchase | External Contracts |
| Lab Equipment | Telephone Equipment - Purchases & Repairs | Computer Consumables | Subscriptions\Membership Fees |
| Spinal Rigid Appliances | Mobile Phones, Bleeps & Two Way Radios | Consumables Building | Estates Recharges |
| Tariff Drugs Recharge | Lease Car Insurance | Consumables Electrical | Disposable assets |
| Drugs Consumables Recharges | Lease Car Hire Charge & Mileage Expenses | Travelling & Subsistence Expenses |  |
| Water Quality Testing | PFI Domestics | Vehicle Maintenance |  |
