# Supplementary material for: The true cost of dialysis modalities: A detailed analysis from a UK renal centre
Source: PLoS One. 2026 Nov 12;21(11):e0362592. doi: 10.1371/journal.pone.0362592 (PMC13759095; doi:10.1371/journal.pone.0362592)
Supplement: Supplementary file 4 [file pone.0362592.s004.docx]

**S4A Table.** Sensitivity analyses of alternative overhead assumptions.

| Modality | 0% overhead (£/patient-year) | 15% overhead (£/patient-year) | 30% overhead (£/patient-year) |
| --- | --- | --- | --- |
| ICHD | 19,690.80 | 22,644.42 | 25,598.04 |
| SHD | 23,197.88 | 26,677.56 | 30,157.24 |
| CAPD | 14,411.23 | 16,572.92 | 18,734.61 |
| CCPD | 32,050.99 | 36,858.64 | 41,666.29 |
| AAPD | 35,725.81 | 41,084.68 | 46,443.55 |
| HHD 3/week | 15,930.54 | 18,320.12 | 20,709.70 |
| HHD 4/week | 21,240.87 | 24,427.00 | 27,613.13 |
| HHD 5/week | 26,551.20 | 30,533.88 | 34,516.56 |

Interpretation: Applying a common overhead rate to all modalities changes absolute costs but not the broad ranking: CAPD and HHD three times weekly remain the two lowest-cost modalities, while CCPD and AAPD remain the two highest-cost modalities in all three scenarios.
