# Supplementary material for: The true cost of dialysis modalities: A detailed analysis from a UK renal centre
Source: PLoS One. 2026 Nov 12;21(11):e0362592. doi: 10.1371/journal.pone.0362592 (PMC13759095; doi:10.1371/journal.pone.0362592)
Supplement: Supplementary file 5 [file pone.0362592.s005.docx]

**HHD cost-allocation sensitivity analysis.** An additional sensitivity analysis examined the effect of increasing HHD treatment frequency under an alternative cost-allocation assumption. The primary analysis cost for HHD delivered three times weekly was retained as the baseline. Non-equipment programme costs were assumed to remain unchanged with increasing treatment frequency, while equipment costs were assumed to increase with each additional dialysis session. The incremental equipment cost per treatment was calculated by dividing annual HHD equipment expenditure (£1,090,869.91) by the total number of HHD sessions (13,520). The same 15% overhead used in the primary HHD analysis was applied to the incremental equipment cost. This analysis represents an alternative modelling scenario and does not replace the primary HHD costing methodology.

**S4B Table. HHD sensitivity analysis assuming unchanged non-equipment costs and frequency-dependent equipment costs**

| HHD frequency | Weekly cost (£) | Cost per session (£) | Annual cost per patient (£) |
| --- | --- | --- | --- |
| 3/week | 352.31 | 117.44 | 18,320.12 |
| 4/week | 445.10 | 111.27 | 23,145.12 |
| 5/week | 537.89 | 107.58 | 27,970.12 |

**Note:** The three-times-weekly HHD cost from the primary analysis was retained as the baseline. For each additional weekly dialysis session, only the equipment component was increased; non-equipment programme costs were assumed to remain unchanged. The same 15% overhead used in the primary analysis was applied to the additional equipment cost. These estimates represent a sensitivity analysis and do not replace the primary HHD results.
