# Supplementary material for: The true cost of dialysis modalities: A detailed analysis from a UK renal centre
Source: PLoS One. 2026 Nov 12;21(11):e0362592. doi: 10.1371/journal.pone.0362592 (PMC13759095; doi:10.1371/journal.pone.0362592)
Supplement: Supplementary file 6 [file pone.0362592.s007.docx]

Dr. Nitin Kolhe

Consultant Nephrologist & Deputy Medical Director and CCIO

University Hospitals of Derby and Burton NHS Foundation Trust

Uttoxeter Road, Derby DE22 3NE

13 September 2026

Professor Cristiana Abbafati

Editor, PLOS ONE

Sapienza University of Rome

Italy

Dear Professor Abbafati,

**Re: Revised manuscript, “The true cost of dialysis modalities: a detailed analysis from a UK renal centre”**

Thank you for the opportunity to revise our manuscript and for the detailed and constructive comments from the Editor and Reviewers. We are pleased to resubmit the revised manuscript for consideration by PLOS ONE.

We have carefully addressed all of the points raised and have substantially revised the manuscript and Supporting Information. All changes in the revised manuscript are tracked to facilitate review. The details are as below:

**Response to Editor and Reviewers**

Manuscript: “The true cost of dialysis modalities: a detailed analysis from a UK renal centre”

We thank the Editor and Reviewers for their detailed and constructive comments. We have substantially revised the manuscript and Supporting Information. All manuscript changes are highlighted in yellow in the accompanying revised files.

**Editor comment 1**

**Comment:** The manuscript uses terms such as cost-effective/cost-effectiveness/economically favourable despite not incorporating clinical outcomes.

**Response:** We agree. This is a provider-perspective micro-costing and financial benchmarking analysis, not a formal cost-effectiveness analysis. We have removed or qualified cost-effectiveness terminology throughout and now distinguish provider cost, BPT benchmark margin and clinical effectiveness.

**Changes made:** Terminology revised throughout the Abstract, Methods, Results, Discussion, tables and Conclusion; BPT comparisons are now described as benchmark margins.

**Editor comment 2**

**Comment:** Further information is required on allocation of shared workforce and other costs.

**Response:** We agree and have retained and clarified the detailed workforce allocation rules in S1 Text. Consultant programmed activities were mapped by clinical activity; administrative and SPA time was proportionally allocated; teaching and acute medicine commitments funded separately were excluded from dialysis; on-call consultant activity was excluded because it predominantly related to medical HDU; resident doctor contributions were allocated by estimated activity proportions; and administrative/KCT inputs were apportioned using documented activity measures.

**Changes made:** The main Methods now points explicitly to the detailed S1 workforce methodology, and the Supplementary Information retains the allocation rules, denominators and treatment of shared activities.

**Editor comment 3**

**Comment:** Explain the 30%/15% overhead rates, possible double counting, and provide sensitivity analysis.

**Response:** The rates are local UHDB institutional overhead assumptions supplied by Finance, not national NHS rates. They are intended to capture residual indirect organisational/infrastructure costs after directly attributable costs have been identified. We reviewed direct cost components to minimise overlap. As the institutional percentage cannot be retrospectively decomposed into each corporate component, we state this limitation explicitly. We added deterministic sensitivity analysis applying common 0%, 15% and 30% overhead rates to all modalities.

**Changes made:** Methods expanded; new S4 Text and S4 Table added. The sensitivity analysis shows that CAPD and three-times-weekly HHD remain the two lowest-cost modalities, while CCPD and AAPD remain the two highest-cost modalities under all three common-overhead scenarios.

**Editor comment 4**

**Comment:** Clarify whether BPT values represent actual or notional reimbursement under the NHS Payment Scheme.

**Response:** We agree. The NHS Payment Scheme replaced the National Tariff Payment System from 1 April 2023 and permits blended payment arrangements. We have therefore treated the 2023–24 BPT values, adjusted for MFF, as standardised financial benchmarks rather than claiming they represent incremental cash reimbursement received by the Trust for every treatment.

**Changes made:** Methods and Discussion revised; 'surplus', 'deficit' and 'financial return' have been replaced or qualified as positive/negative benchmark margins where appropriate.

**Editor comment 5**

**Comment:** The same ICHD total cost is used for AVF and CVC; reconcile the claim that CVC deficits reflect higher consumable costs.

**Response:** We agree. The previous wording was incorrect. The CVC consumable estimate (£37.04/session) is lower than the overall mean (£42.53/session), and access-specific total costs could not be estimated. Differences in AVF/CVC benchmark margins are therefore driven principally by different BPT values, not demonstrated differences in total provider cost.

**Changes made:** Results and Discussion corrected and conclusions about the financial benefit of AVF based on this costing analysis have been moderated.

**Editor comment 6**

**Comment:** Clarify HHD fixed costs and resolve the statement that higher frequency dilutes fixed costs.

**Response:** We agree that the relationship between HHD treatment frequency and programme costs required further clarification. We have retained the original activity-based HHD costing as the primary analysis, with costs of £352.31, £469.75 and £587.19 per patient-week for HHD delivered three, four and five times weekly, respectively. These estimates reflect the primary costing methodology and have not been replaced by the sensitivity analysis.

To examine the effect of an alternative cost-allocation assumption, we added a sensitivity analysis in which the primary three-times-weekly HHD cost (£352.31 per patient-week) was retained as the baseline. Non-equipment programme costs were assumed to remain unchanged as dialysis frequency increased, while equipment costs increased with each additional dialysis session. Annual equipment expenditure (£1,090,869.91) was divided by 13,520 HHD sessions to derive the incremental equipment cost per additional treatment, to which the same 15% overhead used in the primary analysis was applied. Under this alternative scenario, weekly costs were £352.31, £445.10 and £537.89 for three, four and five treatments weekly, corresponding to £117.44, £111.27 and £107.58 per session, respectively. Thus, although weekly costs increased with treatment frequency, the average cost per session decreased under this alternative allocation assumption.

**Changes made:** We clarified the primary HHD costing methodology and added an HHD sensitivity analysis to the Methods, Results and S4 Table. We explicitly describe this as an alternative modelling scenario and do not assume that non-equipment HHD costs are inherently fixed.

**Editor comment 7**

**Response:** We agree that sensitivity analyses were required to assess the effect of key costing assumptions. We have undertaken two relevant sensitivity analyses. First, we tested common overhead rates of 0%, 15% and 30% across dialysis modalities. Second, for HHD, we examined an alternative cost-allocation scenario in which the primary three-times-weekly HHD cost was retained as the baseline, non-equipment programme costs were assumed to remain unchanged with increasing treatment frequency, and equipment costs increased with each additional dialysis session. Under the latter scenario, weekly HHD costs were £352.31, £445.10 and £537.89 for three, four and five treatments weekly, respectively, compared with £352.31, £469.75 and £587.19 in the primary analysis. The corresponding sensitivity-analysis costs per session were £117.44, £111.27 and £107.58. These analyses demonstrate the effect of alternative overhead and HHD cost-allocation assumptions on the estimated provider costs.

We did not undertake a separate numerical workforce sensitivity analysis because workforce allocations were based on documented job plans and activity mapping, and we did not have an empirical basis for specifying alternative allocation percentages. Applying arbitrary percentage changes would therefore have introduced unsupported assumptions.

**Changes made:** The sensitivity-analysis methodology has been expanded in the Methods and Supplementary Methods, with the results reported in the Results and S4 Table. The assumptions and limitations of the HHD sensitivity analysis are explicitly stated.

**Editor comment 8**

**Comment:** Clarify annualisation of equipment/capital, training/setup costs and unused capacity.

**Response:** We have clarified that the analysis used 2023–24 Trust ledger expenditure and supplier invoices. Where annual equipment/service charges were already represented in the ledger, we used the recorded annual expenditure rather than imposing an additional assumed depreciation schedule. Training/setup costs were included only where recorded as attributable 2023–24 expenditure. Unused capacity was not assigned a separate opportunity cost. We now acknowledge that HHD per-patient cost is programme-size dependent.

**Changes made:** New S4 Text added to the Supplementary Information and limitation added to the main manuscript.

**Editor comment 9**

**Comment:** Distinguish Trust-level findings from wider system implications such as transport, autonomy and quality of life.

**Response:** We agree. Transport was not included in the primary costing and therefore cannot be presented as an observed saving. Patient autonomy and quality of life were also not measured.

**Changes made:** Discussion, Implications and Conclusion revised to describe transport reduction only as a potential additional system-level benefit and to avoid claiming unmeasured clinical/patient benefits.

**Editor comment 10**

**Comment:** Correct internal inconsistencies including 'significantly higher', 'HHD was the costliest', and five-times-weekly HHD profitability.

**Response:** We agree and systematically reconciled the narrative with the tables. 'Significantly' has been removed because no inferential comparison was performed. HHD is no longer described as the costliest; CCPD and AAPD have the highest annual costs. Five-times-weekly HHD is consistently described as having a small negative benchmark margin.

**Changes made:** Abstract, Results, Table 4/Table 5 narrative, Discussion and Conclusion corrected.

**Editor comment 11**

**Comment:** Moderate policy claims and emphasise single-centre reproducible framework/external validation.

**Response:** We agree. The conclusions are now framed as a detailed real-world example from one NHS Trust. We explicitly state that the framework should be adapted using local staffing, procurement, estate, programme-size and overhead assumptions and externally validated in other centres.

**Changes made:** Limitations, Implications and Conclusion revised.

**Reviewer 1 – Comment 1**

**Comment:** Use consistent cost units, at least in the abstract.

**Response:** We agree. The revised Abstract now reports annual per-patient costs across modalities to allow direct comparison.

**Changes made:** Abstract Results rewritten using £/patient-year as the principal cross-modality unit.

**Reviewer 1 – Comment 2**

**Comment:** Explain the basis of 15% and 30% overheads.

**Response:** These are local UHDB Finance institutional rates, not national NHS rates. Their purpose, potential overlap and sensitivity have now been described in detail.

**Changes made:** Methods expanded; S4 Text/S4 Table added.

**Reviewer 1 – Comment 3**

**Comment:** Acknowledge that in-centre and home dialysis populations differ in age/complexity.

**Response:** We agree. The study is service-level costing and did not adjust for patient-level case mix.

**Changes made:** A case-mix limitation has been added explicitly.

**Reviewer 1 – Comment 4**

**Comment:** Separate AVF/CVC cost comparisons are not justified when the same aggregate total cost is applied.

**Response:** We agree that the analysis cannot establish access-specific total delivery costs. We retain access categories only to show how the different BPT benchmark values affect the financial benchmark and explicitly state this limitation.

**Changes made:** Access-specific claims have been moderated and the erroneous consumable-cost explanation removed.

**Reviewer 1 – Comment 5**

**Comment:** Explain the large CAPD–CCPD cost difference.

**Response:** The PD estimates were derived from actual supplier invoices and cross-checked with Trust account codes. The difference reflects local modality-specific supplier expenditure and machine-based therapy costs rather than an assumed solution-price differential. We now emphasise that these contract-specific differences may not generalise.

**Changes made:** PD Results and Supplementary Information expanded.

**Reviewer 1 – Comment 6**

**Comment:** Transport costs were omitted and this may bias comparison with home therapies.

**Response:** We agree. Transport was outside the Trust-level primary costing and is now explicitly treated as an unmeasured potential system-level benefit rather than part of the observed modality cost difference.

**Changes made:** Discussion and Conclusion revised.

**Reviewer 1 – Minor**

**Comment:** Define SHD in the abstract.

**Response:** Done.

**Changes made:** 'Satellite haemodialysis (SHD)' is now written in full at first use in the Abstract.

**Reviewer 2 – Comment 1**

**Comment:** Reframe as micro-costing/cost analysis rather than cost-effectiveness.

**Response:** We agree and have done so throughout.

**Changes made:** Terminology revised throughout.

**Reviewer 2 – Comment 2**

**Comment:** Resolve the HHD inconsistency between £1.376 million and £492.48/week.

**Response:** We agree that the previously reported annual HHD programme cost was incorrect. On review of the underlying costing data, the correct direct annual HHD cost was £1,380,652.03 rather than £1,376,093.93. Applying the 15% overhead gives a total annual programme cost of £1,587,749.83. Across 3,224 patient-weeks, this corresponds to a frequency-weighted mean cost of £492.48 per patient-week, consistent with the values reported in Table 4. The primary HHD costs of £352.31, £469.75 and £587.19 per patient-week for three, four and five treatments weekly, respectively, therefore remain unchanged. The manuscript and Supplementary Information have been corrected to report the accurate annual programme cost.

We have additionally undertaken a sensitivity analysis to examine an alternative treatment-frequency cost-allocation assumption. This sensitivity analysis does not replace the primary HHD results.

**Changes made:** The HHD Results and Tables have been reconciled to the original activity-based HHD costing, and the distinction between the primary analysis and the new sensitivity analysis has been made explicit.

**Reviewer 2 – Comment 3**

**Comment:** Correct the interpretation of five-times-weekly HHD.

**Response:** Agreed. Five-times-weekly HHD is now consistently reported as having a small negative benchmark margin.

**Changes made:** Abstract, Results, Tables and Discussion corrected.

**Reviewer 2 – Comment 4**

**Comment:** Provide transparent included/excluded costs and justify overhead assumptions with sensitivity analyses.

**Response:** Done. The Supplementary Information now explains direct versus overhead costs, annual equipment/contract treatment, programme costs, the overhead sensitivity analysis, and the alternative HHD treatment-frequency cost-allocation sensitivity analysis.

**Changes made:** New S4 Text and S4 Table added.

**Reviewer 2 – Comment 5**

**Comment:** Clarify reimbursement and qualify transport/NHS savings claims.

**Response:** Done. BPT is now treated as a standardised benchmark under the 2023–24 NHS Payment Scheme context, and transport is explicitly outside the primary analysis.

**Changes made:** Methods, Discussion and Conclusion revised.

**Journal requirement 1**

**Comment:** Ensure PLOS ONE style requirements and file naming are met.

**Response:** We have prepared revised manuscript and Supporting Information files with clear submission-ready names. Final journal formatting can be applied at upload if required by the submission system.

**Changes made:** Revised files renamed clearly.

**Journal requirement 2**

**Comment:** Include Supporting Information captions at the end of the manuscript and update in-text citations.

**Response:** Done.

**Changes made:** Supporting Information captions S1 Text, S2 Table, S3 Table and new S4 Table have been added at the end of the manuscript.

Thank you for your consideration. We hope that the revised manuscript is now suitable for publication in PLOS ONE.

Yours sincerely,

Dr Nitin Kolhe

On behalf of all authors
