# Supplementary material for: The true cost of dialysis modalities: A detailed analysis from a UK renal centre
Source: PLoS One. 2026 Nov 12;21(11):e0362592. doi: 10.1371/journal.pone.0362592 (PMC13759095; doi:10.1371/journal.pone.0362592)
Supplement: Supplementary file 7 [file pone.0362592.s008.docx]

Response to Academic Editor

Manuscript: *The true cost of dialysis modalities: a detailed analysis from a UK renal centre*

Journal: PLOS ONE

Dear Prof Kaewput,

Thank you for your careful assessment of our revised manuscript and for recognising the improvements in methodological transparency, numerical reporting and interpretation. We appreciate the opportunity to address the remaining points. Our point-by-point responses follow; manuscript line numbers refer to the revised clean version supplied with this response.

**Additional Editor Comment 1 — In-centre haemodialysis expenditure**

**Comment:** The Results report approximately £5.75 million in annual expenditure for 45,157 in-centre haemodialysis sessions, with a final estimated cost of £164.09 per session after a 30% overhead allocation. Please reconcile these figures.

**Response:** Thank you for identifying this discrepancy. We have corrected the pre-overhead annual expenditure to approximately £5.70 million in the Results (lines 227–236). The reported final cost of £164.09 per session includes the 30% institutional overhead uplift. Multiplying £164.09 by 45,157 sessions gives approximately £7.41 million after overhead; dividing this by 1.30 gives approximately £5.70 million before overhead. The earlier figure of £5.75 million was inconsistent with the final per-session cost and has been corrected. Minor differences at the pound level may arise from the rounding of the reported per-session cost.

**Additional Editor Comment 2 —** Home haemodialysis patient numbers and activity

**Comment:** The number of HHD patients varied between 57 and 62, while the costing model uses 62 patients and 13,520 annual sessions. Please clarify whether this represents actual delivered activity, annualised activity or modelled patient-weeks.

**Response:** We agree that this distinction is important. The Results (lines 279–290) now explain that the number of patients receiving HHD fluctuated between 57 and 62 and that 62 patients, the number receiving treatment during most weeks, were used as the model population. The frequency distribution was 15 patients dialysing three times weekly, 20 four times weekly and 27 five times weekly. Applying this distribution over 52 weeks gives (15 × 3 + 20 × 4 + 27 × 5) × 52 = 13,520 modelled annual sessions. Thus, 13,520 is annualised/modelled activity under the specified frequency distribution, rather than a claim that exactly this number of sessions was observed. The total annual programme expenditure was allocated over this modelled activity to derive the reported weekly patient costs. We have retained this distinction in the interpretation of the HHD estimates.

**Additional Editor Comment 3** — Tariff payment units in Table 5

**Comment:** HHD BPT values are weekly, whereas in-centre HD and PD BPT values are per session and per day, respectively. The headings or footnotes should make these units explicit.

**Response:** Thank you. Table 5 now distinguishes “Estimated unit cost” from “BPT (payment unit)” and explicitly displays £/session, £/day or £/week in the relevant rows (Table 5, lines 310–322). The footnote explains that in-centre and satellite haemodialysis are benchmarked per session, peritoneal dialysis per treatment day, and HHD against a fixed weekly BPT irrespective of treatment frequency. Weekly values are derived using the stated treatment schedules, permitting comparison on a common weekly basis. We have also clarified in the Results (lines 326–337) that the HHD benchmark margin declines as frequency increases because its BPT is fixed per week. The figures are described as benchmark margins rather than actual incremental reimbursement.

**Additional Editor Comment 4** — Discussion references

**Comment:** Several Discussion citations do not match the studies described, notably the Value in Health analysis, international HHD comparisons and broader CKD economic evaluations. Please verify and correct citation numbering.

**Response:** We agree and have systematically corrected the citations and reference numbering. The UK integrated home dialysis cost-effectiveness analysis by Erbe and colleagues is now cited as reference 10 (Discussion, lines 352–355); the French comparative HHD study by Rostoker and colleagues as reference 11 (lines 355–357); and the CKD economic burden review by Jha and colleagues as reference 12 (lines 358–361). The subsequent references have been renumbered consistently, the duplicated UK Kidney Association utilities entry has been removed, and the reference list now contains 20 entries. The Welsh costing studies remain correctly cited as references 15 and 16 (lines 385–394).

**Journal requirement — Reference list review**

We have reviewed the reference list for completeness, duplication and correspondence with in-text citations, and have corrected the numbering and removed the duplicate reference.

We thank you again for your constructive comments and hope that these clarifications resolve the remaining concerns.

Yours sincerely,

Nitin V Kolhe, on behalf of all authors
University Hospitals of Derby and Burton NHS Foundation Trust
