# Supplementary material for: The true cost of dialysis modalities: A detailed analysis from a UK renal centre
Source: PLoS One. 2026 Nov 12;21(11):e0362592. doi: 10.1371/journal.pone.0362592 (PMC13759095; doi:10.1371/journal.pone.0362592)
Supplement: Peer review history

# Peer review: Decision Letter 0

**DOI:** 10.1371/journal.pone.0362592.r001

**Authors:** Wisit Kaewput

9 Sep 2026

Dear Dr. Kolhe,

Thank you for submitting your manuscript to PLOS One. After careful consideration, we feel that it has merit but does not fully meet PLOS One’s publication criteria as it currently stands. Therefore, we invite you to submit a revised version of the manuscript that addresses the points raised during the review process.

Please submit your revised manuscript by Nov 08 2026 11:59PM. If you will need more time than this to complete your revisions, please reply to this message or contact the journal office at plosone@plos.org. When you're ready to submit your revision, log on to https://www.editorialmanager.com/pone/ and select the 'Submissions Needing Revision' folder to locate your manuscript file.

- A letter that responds to each point raised by the academic editor and reviewer(s). You should upload this letter as a separate file labeled 'Response to Reviewers'.
- A marked-up copy of your manuscript that highlights changes made to the original version. You should upload this as a separate file labeled 'Revised Manuscript with Track Changes'.
- An unmarked version of your revised paper without tracked changes. You should upload this as a separate file labeled 'Manuscript'.

If you would like to make changes to your financial disclosure, please include your updated statement in your cover letter. Guidelines for resubmitting your figure files are available below the reviewer comments at the end of this letter.

If applicable, we recommend that you deposit your laboratory protocols in protocols.io to enhance the reproducibility of your results. Protocols.io assigns your protocol its own identifier (DOI) so that it can be cited independently in the future. For instructions see: https://journals.plos.org/plosone/s/submission-guidelines#loc-laboratory-protocols. Additionally, PLOS One offers an option for publishing peer-reviewed Lab Protocol articles, which describe protocols hosted on protocols.io. Read more information on sharing protocols at https://plos.org/protocols?utm_medium=editorial-email&utm_source=authorletters&utm_campaign=protocols.

As the corresponding author, your ORCID iD is verified in the submission system and will appear in the published article. PLOS supports the use of ORCID, and we encourage all coauthors to register for an ORCID iD and use it as well. Please encourage your coauthors to verify their ORCID iD within the submission system before final acceptance, as unverified ORCID iDs will not appear in the published article. *Only*  the individual author can complete the verification step; PLOS staff *cannot*  verify ORCID iDs on behalf of authors.

We look forward to receiving your revised manuscript.

Kind regards,

Wisit Kaewput, MD, FRCP, FRCPSG, FASN, FAcadMEd

Academic Editor

PLOS One

Journal Requirements:

When submitting your revision, we need you to address these additional requirements.

1. Please ensure that your manuscript meets PLOS ONE's style requirements, including those for file naming. The PLOS ONE style templates can be found at

https://journals.plos.org/plosone/s/file?id=wjVg/PLOSOne_formatting_sample_main_body.pdf and https://journals.plos.org/plosone/s/file?id=ba62/PLOSOne_formatting_sample_title_authors_affiliations.pdf

2. Please include captions for your Supporting Information files at the end of your manuscript, and update any in-text citations to match accordingly. Please see our Supporting Information guidelines for more information: http://journals.plos.org/plosone/s/supporting-information.

3. If the reviewer comments include a recommendation to cite specific previously published works, please review and evaluate these publications to determine whether they are relevant and should be cited. There is no requirement to cite these works unless the editor has indicated otherwise.

Additional Editor Comments :

The manuscript addresses an important and policy-relevant topic, namely the real-world costs of different dialysis modalities within an NHS renal service. The use of financial ledger data, workforce allocation, consumable expenditure, infrastructure costs, and reimbursement information is a strength and has the potential to provide useful information for service planning. However, several methodological and interpretative issues require clarification and, in some areas, additional analysis before the conclusions can be fully supported. I therefore recommend major revision.

1. The manuscript repeatedly uses terms such as “cost-effective,” “cost-effectiveness,” and “economically favourable.” However, the analysis primarily compares service-delivery costs with reimbursement tariffs and does not incorporate measures of clinical effectiveness, quality-adjusted life-years, survival, hospitalisation, patient-reported outcomes, or other health outcomes. In the conventional health-economic sense, the current study would therefore be more appropriately described as a provider-perspective micro-costing and financial-margin analysis rather than a formal cost-effectiveness analysis. I suggest that the authors revise the terminology throughout the title, abstract, methods, discussion, and conclusions to more accurately reflect the study design and avoid implying assessment of clinical or health-economic effectiveness that was not undertaken.

2. A key component of the analysis is the allocation of workforce and other shared costs to individual dialysis modalities. The manuscript states that staff costs were allocated according to job plans, estimated time contribution, and activity mapping. Further information would help readers understand and reproduce this approach. In particular, the authors should explain how shared clinical activities, administrative duties, on-call responsibilities, teaching, outpatient activities, and cross-cover were allocated. Because workforce expenditure represents a substantial component of dialysis costs, the assumptions used for these allocations could materially influence the modality-specific estimates. A clearer description of allocation rules, denominators, unit costs, and treatment of shared resources would strengthen the methodological transparency of the study.

3. The authors apply a 30% overhead uplift to haemodialysis delivered at Royal Derby Hospital and 15% to community, satellite, and home therapies. Because these adjustments are relatively large and directly affect the estimated differences between modalities, the manuscript should provide a clearer explanation of how these percentages were derived and which specific costs they represent. The authors should also clarify whether any components incorporated within these overhead rates may already be captured elsewhere in the costing model. Ideally, a sensitivity analysis using alternative plausible overhead assumptions should be provided to demonstrate whether the relative ranking of dialysis modalities remains stable.

4. The analysis calculates positive or negative margins by comparing estimated costs with Best Practice Tariff reimbursement adjusted for the Market Forces Factor. However, the manuscript also notes that NHS payment arrangements transitioned from the National Tariff Payment System to the NHS Payment Scheme, with blended payment mechanisms. The authors should therefore clarify how reimbursement operated at the study Trust during the 2023–24 financial year and whether the tariff values used in the analysis represent actual or notional reimbursement for the relevant dialysis activity. This distinction is important when describing the calculated differences as “surplus,” “deficit,” or “financial return.” If the tariff comparison is intended primarily as a benchmarking exercise, this should be explicitly stated and the terminology adjusted accordingly.

5. The manuscript reports the same final mean cost of £164.09 for all in-centre haemodialysis access categories because granular staffing and operational costs could not be separately allocated according to vascular access. Consequently, the observed differences in financial margin between AVF and CVC dialysis appear to be driven substantially by differences in reimbursement rather than independently estimated differences in total delivery cost. In addition, the statement that CVC dialysis generated deficits primarily because of higher consumable costs should be reconciled with Table 2, where the reported CVC consumable cost appears lower than the overall average. The authors should clarify these calculations and moderate conclusions regarding the financial benefit of increasing AVF use unless supported by access-specific total cost estimates.

6. Equipment represents the largest component of HHD expenditure, suggesting that a substantial proportion of programme costs may be fixed. However, the reported weekly costs appear to increase almost proportionally with treatment frequency across three, four, and five sessions per week. The authors should explain how fixed equipment and programme costs were allocated across treatment frequencies and how the per-session HHD cost was derived. The statement that higher treatment frequency results in “greater fixed-cost dilution and improved efficiency” should also be reconciled with the reported cost calculations and with the finding that five-times-weekly HHD becomes cost-negative relative to the stated tariff. A clearer separation of fixed and variable HHD costs would make these findings easier to interpret.

7. The manuscript currently reports point estimates of costs and margins, often to a high degree of numerical precision. However, several inputs depend on estimated allocation of staff time, shared resources, overhead assumptions, and programme-level costs. The authors should consider providing deterministic sensitivity analyses for the principal assumptions, particularly workforce allocation, overhead rates, and HHD fixed-cost allocation. A full probabilistic economic model may not be necessary for this descriptive costing study, but reasonable scenario analyses would help readers determine whether conclusions are robust to plausible changes in key assumptions. This would be especially useful for modalities where the estimated positive or negative financial margin is relatively small.

8. The different dialysis modalities have substantially different cost structures. In-centre haemodialysis is particularly dependent on staffing and estate utilisation, whereas HHD includes substantial equipment and programme costs, and PD costs are largely derived from supplier expenditure and treatment days. The authors should clarify how capital and equipment costs were annualised, whether depreciation or equipment lifespan was incorporated, how training or programme setup costs were treated, and how unused capacity was handled. This is particularly relevant for HHD, where only 62 patients were included and the per-patient cost may depend on programme size. Providing this information would also improve interpretation of the potential economic consequences of expanding home dialysis services.

9. The study primarily evaluates costs from the perspective of a single NHS Trust, and the authors acknowledge that transport and broader system-level costs were not incorporated into the main costing analysis. The discussion and conclusion should therefore distinguish between findings directly demonstrated by the present analysis and potential wider health-system implications. For example, reduced transport expenditure with home dialysis is plausible and relevant, but because transport was not incorporated into the primary calculations, this should be presented as a potential additional system-level benefit rather than as an observed result of this study. Similarly, conclusions regarding patient autonomy, quality of life, or broader clinical benefits should be clearly distinguished from the economic findings presented here.

10. There are several places where the narrative does not appear fully consistent with the tabulated findings. For example, CCPD and AAPD are described as having “significantly higher” costs although no statistical comparison appears to have been performed. Table 5 identifies CAPD and lower-frequency HHD as among the least expensive modalities, whereas the accompanying text states that “Home haemodialysis was the costliest,” despite higher annual costs being reported for CCPD and AAPD. There are also varying descriptions of the profitability of HHD across different sections, particularly for the five-times-weekly regimen. These discrepancies should be systematically reviewed and corrected so that the abstract, results, tables, discussion, and conclusions consistently reflect the same findings.

11. This is a detailed single-centre study conducted within a Trust with a particular staffing structure, procurement system, PFI arrangement, modality distribution, overhead structure, and local Market Forces Factor. These characteristics may differ across NHS organisations. The manuscript appropriately acknowledges this limitation, but some statements regarding policy development and prioritisation of home dialysis pathways are broader than the available data support. The authors should present the current findings primarily as a detailed real-world example and potentially reproducible costing framework. The value of the study would be further strengthened by discussing how the model might be adapted or externally validated in other NHS centres.

In summary, this is a potentially valuable real-world micro-costing study addressing an important issue in dialysis service delivery. The detailed use of operational and financial data is a notable strength. However, clarification of the economic framework, greater transparency regarding cost allocation, appropriate interpretation of tariffs, sensitivity analyses around key assumptions, reconciliation of several internal inconsistencies, and more cautious framing of the conclusions are needed. These issues appear addressable through substantial revision and would considerably strengthen the validity and interpretability of the manuscript. I therefore recommend major revision.

[Note: HTML markup is below. Please do not edit.]

Reviewers' comments:

Reviewer's Responses to Questions

**Comments to the Author**

1. Is the manuscript technically sound, and do the data support the conclusions?

Reviewer #1: Partly

Reviewer #2: Partly

**********

2. Has the statistical analysis been performed appropriately and rigorously?

Reviewer #1: Yes

Reviewer #2: Yes

**********

3. Have the authors made all data underlying the findings in their manuscript fully available?

The PLOS Data policy

Reviewer #1: No

Reviewer #2: Yes

**********

4. Is the manuscript presented in an intelligible fashion and written in standard English?

Reviewer #1: Yes

Reviewer #2: Yes

**********

Reviewer #1: Thank you for the opportunity to review this interesting manuscript. As dialysis treatment is associated with substantial costs, it is of great importance to conduct the most reliable possible comparisons of the costs of different dialysis modalities in order to support rational allocation of healthcare resources. This study provides a thorough assessment of these costs; however, I would like to raise several comments regarding the manuscript:

• Cost estimates are reported using varying units, including costs per day, week, year, and treatment session. At a minimum, the abstract should use a consistent unit throughout to facilitate clear comparisons.

• Overhead costs of 15% and 30% were added to the cost calculations; however, the manuscript does not explain the basis for these percentages or how they were determined.

• When comparing actual treatment costs, it should be acknowledged that the patient populations receiving in-center hemodialysis and home dialysis differ substantially. These differences are likely to influence costs, as the care of older and more medically complex patients is generally more expensive regardless of dialysis modality.

• In my view, it is not justified to present separate cost comparisons for hemodialysis performed via an arteriovenous fistula versus a catheter if these access types cannot be distinguished in the cost calculations and the same aggregate cost estimate is applied to both.

• The study demonstrated a surprisingly large difference in costs between CAPD and CCPD. I would expect the costs of peritoneal dialysis solutions to be broadly similar. Although CCPD undoubtedly requires somewhat greater staff resources and incurs additional equipment costs, a more than two-fold difference in overall cost appears unexpected. The underlying reasons for this discrepancy should be explained in greater detail. While contracts for peritoneal dialysis may vary between hospitals, equipment costs of this magnitude are nevertheless surprising and may limit the generalizability of the findings.

• As noted in the manuscript, patient transportation costs constitute a substantial component of the overall costs of dialysis care. However, these costs were not included in the present analysis. This omission introduces a clear bias when comparing home dialysis modalities with in-center hemodialysis.

• Minor comment: The abbreviation SHD is not defined in the abstract and should be written out in full when first mentioned.

Reviewer #2: Thank you to the authors for their efforts. The study addresses a clinically relevant question and uses a potentially valuable dataset. The detailed use of Trust financial ledgers and workforce mapping is a particular strength. However, the manuscript currently overstates the findings, and several numerical and methodological issues—particularly regarding HHD—need clarification.

1. Reframe the study as a micro-costing/cost analysis rather than “cost-effectiveness” unless clinical outcomes or QALYs are incorporated.

2. Resolve the HHD numerical inconsistency between the reported £1.376 million annual expenditure and £492.48/week frequency-weighted cost.

3. Correct the contradictory interpretation of five-times-weekly HHD, which is reported to have a negative reimbursement margin (−£13.95/week).

4. Provide a transparent modality-specific breakdown of included and excluded costs, and justify the 15%/30% overhead assumptions, preferably with sensitivity analyses.

5. Clarify the reimbursement methodology and remove or qualify claims regarding transport savings and overall NHS cost reduction, as transport and broader system costs were not directly analysed.

**********

what does this mean?). If published, this will include your full peer review and any attached files.

If you choose “no”, your identity will remain anonymous but your review may still be made public.

**Do you want your identity to be public for this peer review?**  For information about this choice, including consent withdrawal, please see our Privacy Policy

Reviewer #1: No

Reviewer #2: No

**********

[NOTE: If reviewer comments were submitted as an attachment file, they will be attached to this email and accessible via the submission site. Please log into your account, locate the manuscript record, and check for the action link "View Attachments". If this link does not appear, there are no attachment files.]

To ensure your figures meet our technical requirements, please review our figure guidelines: https://journals.plos.org/plosone/s/figures

You may also use PLOS’s free figure tool, NAAS, to help you prepare publication quality figures: https://journals.plos.org/plosone/s/figures#loc-tools-for-figure-preparation.

NAAS will assess whether your figures meet our technical requirements by comparing each figure against our figure specifications.

# Author comment: Author response to Decision Letter 1

**DOI:** 10.1371/journal.pone.0362592.r002

15 Sep 2026

Response to Editor and Reviewers

Manuscript: “The true cost of dialysis modalities: a detailed analysis from a UK renal centre”

We thank the Editor and Reviewers for their detailed and constructive comments. We have substantially revised the manuscript and Supporting Information. All manuscript changes are highlighted in yellow in the accompanying revised files.

Editor comment 1

Comment: The manuscript uses terms such as cost-effective/cost-effectiveness/economically favourable despite not incorporating clinical outcomes.

Response: We agree. This is a provider-perspective micro-costing and financial benchmarking analysis, not a formal cost-effectiveness analysis. We have removed or qualified cost-effectiveness terminology throughout and now distinguish provider cost, BPT benchmark margin and clinical effectiveness.

Changes made: Terminology revised throughout the Abstract, Methods, Results, Discussion, tables and Conclusion; BPT comparisons are now described as benchmark margins.

Editor comment 2

Comment: Further information is required on allocation of shared workforce and other costs.

Response: We agree and have retained and clarified the detailed workforce allocation rules in S1 Text. Consultant programmed activities were mapped by clinical activity; administrative and SPA time was proportionally allocated; teaching and acute medicine commitments funded separately were excluded from dialysis; on-call consultant activity was excluded because it predominantly related to medical HDU; resident doctor contributions were allocated by estimated activity proportions; and administrative/KCT inputs were apportioned using documented activity measures.

Changes made: The main Methods now points explicitly to the detailed S1 workforce methodology, and the Supplementary Information retains the allocation rules, denominators and treatment of shared activities.

Editor comment 3

Comment: Explain the 30%/15% overhead rates, possible double counting, and provide sensitivity analysis.

Response: The rates are local UHDB institutional overhead assumptions supplied by Finance, not national NHS rates. They are intended to capture residual indirect organisational/infrastructure costs after directly attributable costs have been identified. We reviewed direct cost components to minimise overlap. As the institutional percentage cannot be retrospectively decomposed into each corporate component, we state this limitation explicitly. We added deterministic sensitivity analysis applying common 0%, 15% and 30% overhead rates to all modalities.

Changes made: Methods expanded; new S4 Text and S4 Table added. The sensitivity analysis shows that CAPD and three-times-weekly HHD remain the two lowest-cost modalities, while CCPD and AAPD remain the two highest-cost modalities under all three common-overhead scenarios.

Editor comment 4

Comment: Clarify whether BPT values represent actual or notional reimbursement under the NHS Payment Scheme.

Response: We agree. The NHS Payment Scheme replaced the National Tariff Payment System from 1 April 2023 and permits blended payment arrangements. We have therefore treated the 2023–24 BPT values, adjusted for MFF, as standardised financial benchmarks rather than claiming they represent incremental cash reimbursement received by the Trust for every treatment.

Changes made: Methods and Discussion revised; 'surplus', 'deficit' and 'financial return' have been replaced or qualified as positive/negative benchmark margins where appropriate.

Editor comment 5

Comment: The same ICHD total cost is used for AVF and CVC; reconcile the claim that CVC deficits reflect higher consumable costs.

Response: We agree. The previous wording was incorrect. The CVC consumable estimate (£37.04/session) is lower than the overall mean (£42.53/session), and access-specific total costs could not be estimated. Differences in AVF/CVC benchmark margins are therefore driven principally by different BPT values, not demonstrated differences in total provider cost.

Changes made: Results and Discussion corrected and conclusions about the financial benefit of AVF based on this costing analysis have been moderated.

Editor comment 6

Comment: Clarify HHD fixed costs and resolve the statement that higher frequency dilutes fixed costs.

Response: We agree that the relationship between HHD treatment frequency and programme costs required further clarification. We have retained the original activity-based HHD costing as the primary analysis, with costs of £352.31, £469.75 and £587.19 per patient-week for HHD delivered three, four and five times weekly, respectively. These estimates reflect the primary costing methodology and have not been replaced by the sensitivity analysis.

To examine the effect of an alternative cost-allocation assumption, we added a sensitivity analysis in which the primary three-times-weekly HHD cost (£352.31 per patient-week) was retained as the baseline. Non-equipment programme costs were assumed to remain unchanged as dialysis frequency increased, while equipment costs increased with each additional dialysis session. Annual equipment expenditure (£1,090,869.91) was divided by 13,520 HHD sessions to derive the incremental equipment cost per additional treatment, to which the same 15% overhead used in the primary analysis was applied. Under this alternative scenario, weekly costs were £352.31, £445.10 and £537.89 for three, four and five treatments weekly, corresponding to £117.44, £111.27 and £107.58 per session, respectively. Thus, although weekly costs increased with treatment frequency, the average cost per session decreased under this alternative allocation assumption.

Changes made: We clarified the primary HHD costing methodology and added an HHD sensitivity analysis to the Methods, Results and S4 Table. We explicitly describe this as an alternative modelling scenario and do not assume that non-equipment HHD costs are inherently fixed.

Editor comment 7

Response: We agree that sensitivity analyses were required to assess the effect of key costing assumptions. We have undertaken two relevant sensitivity analyses. First, we tested common overhead rates of 0%, 15% and 30% across dialysis modalities. Second, for HHD, we examined an alternative cost-allocation scenario in which the primary three-times-weekly HHD cost was retained as the baseline, non-equipment programme costs were assumed to remain unchanged with increasing treatment frequency, and equipment costs increased with each additional dialysis session. Under the latter scenario, weekly HHD costs were £352.31, £445.10 and £537.89 for three, four and five treatments weekly, respectively, compared with £352.31, £469.75 and £587.19 in the primary analysis. The corresponding sensitivity-analysis costs per session were £117.44, £111.27 and £107.58. These analyses demonstrate the effect of alternative overhead and HHD cost-allocation assumptions on the estimated provider costs.

We did not undertake a separate numerical workforce sensitivity analysis because workforce allocations were based on documented job plans and activity mapping, and we did not have an empirical basis for specifying alternative allocation percentages. Applying arbitrary percentage changes would therefore have introduced unsupported assumptions.

Changes made: The sensitivity-analysis methodology has been expanded in the Methods and Supplementary Methods, with the results reported in the Results and S4 Table. The assumptions and limitations of the HHD sensitivity analysis are explicitly stated.

Editor comment 8

Comment: Clarify annualisation of equipment/capital, training/setup costs and unused capacity.

Response: We have clarified that the analysis used 2023–24 Trust ledger expenditure and supplier invoices. Where annual equipment/service charges were already represented in the ledger, we used the recorded annual expenditure rather than imposing an additional assumed depreciation schedule. Training/setup costs were included only where recorded as attributable 2023–24 expenditure. Unused capacity was not assigned a separate opportunity cost. We now acknowledge that HHD per-patient cost is programme-size dependent.

Changes made: New S4 Text added to the Supplementary Information and limitation added to the main manuscript.

Editor comment 9

Comment: Distinguish Trust-level findings from wider system implications such as transport, autonomy and quality of life.

Response: We agree. Transport was not included in the primary costing and therefore cannot be presented as an observed saving. Patient autonomy and quality of life were also not measured.

Changes made: Discussion, Implications and Conclusion revised to describe transport reduction only as a potential additional system-level benefit and to avoid claiming unmeasured clinical/patient benefits.

Editor comment 10

Comment: Correct internal inconsistencies including 'significantly higher', 'HHD was the costliest', and five-times-weekly HHD profitability.

Response: We agree and systematically reconciled the narrative with the tables. 'Significantly' has been removed because no inferential comparison was performed. HHD is no longer described as the costliest; CCPD and AAPD have the highest annual costs. Five-times-weekly HHD is consistently described as having a small negative benchmark margin.

Changes made: Abstract, Results, Table 4/Table 5 narrative, Discussion and Conclusion corrected.

Editor comment 11

Comment: Moderate policy claims and emphasise single-centre reproducible framework/external validation.

Response: We agree. The conclusions are now framed as a detailed real-world example from one NHS Trust. We explicitly state that the framework should be adapted using local staffing, procurement, estate, programme-size and overhead assumptions and externally validated in other centres.

Changes made: Limitations, Implications and Conclusion revised.

Reviewer 1 – Comment 1

Comment: Use consistent cost units, at least in the abstract.

Response: We agree. The revised Abstract now reports annual per-patient costs across modalities to allow direct comparison.

Changes made: Abstract Results rewritten using £/patient-year as the principal cross-modality unit.

Reviewer 1 – Comment 2

Comment: Explain the basis of 15% and 30% overheads.

Response: These are local UHDB Finance institutional rates, not national NHS rates. Their purpose, potential overlap and sensitivity have now been described in detail.

Changes made: Methods expanded; S4 Text/S4 Table added.

Reviewer 1 – Comment 3

Comment: Acknowledge that in-centre and home dialysis populations differ in age/complexity.

Response: We agree. The study is service-level costing and did not adjust for patient-level case mix.

Changes made: A case-mix limitation has been added explicitly.

Reviewer 1 – Comment 4

Comment: Separate AVF/CVC cost comparisons are not justified when the same aggregate total cost is applied.

Response: We agree that the analysis cannot establish access-specific total delivery costs. We retain access categories only to show how the different BPT benchmark values affect the financial benchmark and explicitly state this limitation.

Changes made: Access-specific claims have been moderated and the erroneous consumable-cost explanation removed.

Reviewer 1 – Comment 5

Comment: Explain the large CAPD–CCPD cost difference.

Response: The PD estimates were derived from actual supplier invoices and cross-checked with Trust account codes. The difference reflects local modality-specific supplier expenditure and machine-based therapy costs rather than an assumed solution-price differential. We now emphasise that these contract-specific differences may not generalise.

Changes made: PD Results and Supplementary Information expanded.

Reviewer 1 – Comment 6

Comment: Transport costs were omitted and this may bias comparison with home therapies.

Response: We agree. Transport was outside the Trust-level primary costing and is now explicitly treated as an unmeasured potential system-level benefit rather than part of the observed modality cost difference.

Changes made: Discussion and Conclusion revised.

Reviewer 1 – Minor

Comment: Define SHD in the abstract.

Response: Done.

Changes made: 'Satellite haemodialysis (SHD)' is now written in full at first use in the Abstract.

Reviewer 2 – Comment 1

Comment: Reframe as micro-costing/cost analysis rather than cost-effectiveness.

Response: We agree and have done so throughout.

Changes made: Terminology revised throughout.

Reviewer 2 – Comment 2

Comment: Resolve the HHD inconsistency between £1.376 million and £492.48/week.

Response: We agree that the previously reported annual HHD programme cost was incorrect. On review of the underlying costing data, the correct direct annual HHD cost was £1,380,652.03 rather than £1,376,093.93. Applying the 15% overhead gives a total annual programme cost of £1,587,749.83. Across 3,224 patient-weeks, this corresponds to a frequency-weighted mean cost of £492.48 per patient-week, consistent with the values reported in Table 4. The primary HHD costs of £352.31, £469.75 and £587.19 per patient-week for three, four and five treatments weekly, respectively, therefore remain unchanged. The manuscript and Supplementary Information have been corrected to report the accurate annual programme cost.

We have additionally undertaken a sensitivity analysis to examine an alternative treatment-frequency cost-allocation assumption. This sensitivity analysis does not replace the primary HHD results.

Changes made: The HHD Results and Tables have been reconciled to the original activity-based HHD costing, and the distinction between the primary analysis and the new sensitivity analysis has been made explicit.

Reviewer 2 – Comment 3

Comment: Correct the interpretation of five-times-weekly HHD.

Response: Agreed. Five-times-weekly HHD is now consistently reported as having a small negative benchmark margin.

Changes made: Abstract, Results, Tables and Discussion corrected.

Reviewer 2 – Comment 4

Comment: Provide transparent included/excluded costs and justify overhead assumptions with sensitivity analyses.

Response: Done. The Supplementary Information now explains direct versus overhead costs, annual equipment/contract treatment, programme costs, the overhead sensitivity analysis, and the alternative HHD treatment-frequency cost-allocation sensitivity analysis.

Changes made: New S4 Text and S4 Table added.

Reviewer 2 – Comment 5

Comment: Clarify reimbursement and qualify transport/NHS savings claims.

Response: Done. BPT is now treated as a standardised benchmark under the 2023–24 NHS Payment Scheme context, and transport is explicitly outside the primary analysis.

Changes made: Methods, Discussion and Conclusion revised.

Journal requirement 1

Comment: Ensure PLOS ONE style requirements and file naming are met.

Response: We have prepared revised manuscript and Supporting Information files with clear submission-ready names. Final journal formatting can be applied at upload if required by the submission system.

Changes made: Revised files renamed clearly.

Journal requirement 2

Comment: Include Supporting Information captions at the end of the manuscript and update in-text citations.

Response: Done.

Changes made: Supporting Information captions S1 Text, S2 Table, S3 Table and new S4 Table have been added at the end of the manuscript.

Attachment Submitted filename: PLOS_ONE_response_to_reviewers_dialysis_costing_FINAL.docx

# Peer review: Decision Letter 1

**DOI:** 10.1371/journal.pone.0362592.r003

**Authors:** Wisit Kaewput

23 Oct 2026

Dear Dr. Kolhe,

Thank you for submitting your manuscript to PLOS One. After careful consideration, we feel that it has merit but does not fully meet PLOS One’s publication criteria as it currently stands. Therefore, we invite you to submit a revised version of the manuscript that addresses the points raised during the review process.

Please submit your revised manuscript by Nov 22 2026 11:59PM. If you will need more time than this to complete your revisions, please reply to this message or contact the journal office at plosone@plos.org. When you're ready to submit your revision, log on to https://www.editorialmanager.com/pone/ and select the 'Submissions Needing Revision' folder to locate your manuscript file.

- A letter that responds to each point raised by the academic editor and reviewer(s). You should upload this letter as a separate file labeled 'Response to Reviewers'.
- A marked-up copy of your manuscript that highlights changes made to the original version. You should upload this as a separate file labeled 'Revised Manuscript with Track Changes'.
- An unmarked version of your revised paper without tracked changes. You should upload this as a separate file labeled 'Manuscript'.

If you would like to make changes to your financial disclosure, please include your updated statement in your cover letter. Guidelines for resubmitting your figure files are available below the reviewer comments at the end of this letter.

If applicable, we recommend that you deposit your laboratory protocols in protocols.io to enhance the reproducibility of your results. Protocols.io assigns your protocol its own identifier (DOI) so that it can be cited independently in the future. For instructions see: https://journals.plos.org/plosone/s/submission-guidelines#loc-laboratory-protocols. Additionally, PLOS One offers an option for publishing peer-reviewed Lab Protocol articles, which describe protocols hosted on protocols.io. Read more information on sharing protocols at https://plos.org/protocols?utm_medium=editorial-email&utm_source=authorletters&utm_campaign=protocols.

As the corresponding author, your ORCID iD is verified in the submission system and will appear in the published article. PLOS supports the use of ORCID, and we encourage all coauthors to register for an ORCID iD and use it as well. Please encourage your coauthors to verify their ORCID iD within the submission system before final acceptance, as unverified ORCID iDs will not appear in the published article. *Only*  the individual author can complete the verification step; PLOS staff *cannot*  verify ORCID iDs on behalf of authors.

We look forward to receiving your revised manuscript.

Kind regards,

Wisit Kaewput, MD, FRCP, FRCPSG, FASN, FAcadMEd

Academic Editor

PLOS One

Journal Requirements:

1. If the reviewer comments include a recommendation to cite specific previously published works, please review and evaluate these publications to determine whether they are relevant and should be cited. There is no requirement to cite these works unless the editor has indicated otherwise.

2. Please review your reference list to ensure that it is complete and correct. If you have cited papers that have been retracted, please include the rationale for doing so in the manuscript text, or remove these references and replace them with relevant current references. Any changes to the reference list should be mentioned in the rebuttal letter that accompanies your revised manuscript. If you need to cite a retracted article, indicate the article’s retracted status in the References list and also include a citation and full reference for the retraction notice.

**Additional Editor Comments:**

The authors have made substantial efforts to address the concerns raised during the previous round of review. The revised manuscript is considerably improved in terms of methodological transparency, numerical presentation, and interpretation of the findings. In particular, reframing the study as a provider-perspective micro-costing and financial benchmarking analysis, rather than a formal cost-effectiveness evaluation, is appropriate. The additional sensitivity analyses and clarification of institutional overhead allocation, home haemodialysis costing assumptions, and vascular-access-related benchmark margins have strengthened the manuscript.

Overall, I consider that the major methodological concerns have been adequately addressed and that the manuscript is now approaching suitability for publication. However, a few minor issues should be clarified or corrected to ensure numerical consistency and accuracy of reporting.

1. In the Results section, the authors report approximately £5.75 million in annual expenditure for in-centre haemodialysis, comprising 45,157 dialysis sessions, with a final estimated cost of £164.09 per session following a 30% overhead allocation. However, back-calculation from the reported per-session cost yields a pre-overhead expenditure of approximately £5.70 million. Although this difference is relatively small, the authors should reconcile these figures or clarify whether they reflect different expenditure components, exclusions, or rounding conventions.

2. The revised manuscript reports that the number of patients receiving home haemodialysis varied between 57 and 62 during the study period, whereas the frequency-based costing model uses 62 patients and 13,520 annual sessions. The authors should briefly clarify whether the calculations represent actual delivered activity, annualised treatment activity, or modelled patient-weeks. This clarification would improve reproducibility and ensure that the reported annual programme costs and weekly per-patient estimates are interpreted appropriately.

3. Table 5 provides a useful summary of costs and benchmark margins across dialysis modalities. However, the Best Practice Tariff values for home haemodialysis are expressed on a weekly basis, whereas those for in-centre haemodialysis and peritoneal dialysis are expressed per session and per day, respectively. The table headings or footnotes should explicitly distinguish these units to avoid potentially misleading comparisons between modalities.

4. Several citations in the Discussion appear inconsistent with the corresponding reference list. In particular, the references cited for the Value in Health analysis, international comparative costing studies, and broader chronic kidney disease economic evaluations do not appear to correspond consistently to the studies described in the text. The authors should carefully verify these citations, correct any numbering errors, and ensure that the referenced publications directly support the associated statements.

[Note: HTML markup is below. Please do not edit.]

Reviewers' comments:

Reviewer's Responses to Questions

**Comments to the Author**

Reviewer #1: All comments have been addressed

**********

2. Is the manuscript technically sound, and do the data support the conclusions?

Reviewer #1: Yes

**********

3. Has the statistical analysis been performed appropriately and rigorously?

Reviewer #1: Yes

**********

4. Have the authors made all data underlying the findings in their manuscript fully available?

The PLOS Data policy

Reviewer #1: Yes

**********

5. Is the manuscript presented in an intelligible fashion and written in standard English?

Reviewer #1: Yes

**********

Reviewer #1: Thank you for this revised version. All my comments have now been addressed. I believe the manuscript has improved markedly as a result of the changes made and is now acceptable for publication.

**********

what does this mean?). If published, this will include your full peer review and any attached files.

If you choose “no”, your identity will remain anonymous but your review may still be made public.

**Do you want your identity to be public for this peer review?**  For information about this choice, including consent withdrawal, please see our Privacy Policy

Reviewer #1: No

**********

[NOTE: If reviewer comments were submitted as an attachment file, they will be attached to this email and accessible via the submission site. Please log into your account, locate the manuscript record, and check for the action link "View Attachments". If this link does not appear, there are no attachment files.]

To ensure your figures meet our technical requirements, please review our figure guidelines: https://journals.plos.org/plosone/s/figures

You may also use PLOS’s free figure tool, NAAS, to help you prepare publication quality figures: https://journals.plos.org/plosone/s/figures#loc-tools-for-figure-preparation.

NAAS will assess whether your figures meet our technical requirements by comparing each figure against our figure specifications.

# Author comment: Author response to Decision Letter 2

**DOI:** 10.1371/journal.pone.0362592.r004

25 Oct 2026

Response to Academic Editor

Manuscript: The true cost of dialysis modalities: a detailed analysis from a UK renal centre

Journal: PLOS ONE

Dear Prof Kaewput,

Thank you for your careful assessment of our revised manuscript and for recognising the improvements in methodological transparency, numerical reporting and interpretation. We appreciate the opportunity to address the remaining points. Our point-by-point responses follow; manuscript line numbers refer to the revised clean version supplied with this response.

Additional Editor Comment 1 — In-centre haemodialysis expenditure

Comment: The Results report approximately £5.75 million in annual expenditure for 45,157 in-centre haemodialysis sessions, with a final estimated cost of £164.09 per session after a 30% overhead allocation. Please reconcile these figures.

Response: Thank you for identifying this discrepancy. We have corrected the pre-overhead annual expenditure to approximately £5.70 million in the Results (lines 227–236). The reported final cost of £164.09 per session includes the 30% institutional overhead uplift. Multiplying £164.09 by 45,157 sessions gives approximately £7.41 million after overhead; dividing this by 1.30 gives approximately £5.70 million before overhead. The earlier figure of £5.75 million was inconsistent with the final per-session cost and has been corrected. Minor differences at the pound level may arise from the rounding of the reported per-session cost.

Additional Editor Comment 2 — Home haemodialysis patient numbers and activity

Comment: The number of HHD patients varied between 57 and 62, while the costing model uses 62 patients and 13,520 annual sessions. Please clarify whether this represents actual delivered activity, annualised activity or modelled patient-weeks.

Response: We agree that this distinction is important. The Results (lines 279–290) now explain that the number of patients receiving HHD fluctuated between 57 and 62 and that 62 patients, the number receiving treatment during most weeks, were used as the model population. The frequency distribution was 15 patients dialysing three times weekly, 20 four times weekly and 27 five times weekly. Applying this distribution over 52 weeks gives (15 × 3 + 20 × 4 + 27 × 5) × 52 = 13,520 modelled annual sessions. Thus, 13,520 is annualised/modelled activity under the specified frequency distribution, rather than a claim that exactly this number of sessions was observed. The total annual programme expenditure was allocated over this modelled activity to derive the reported weekly patient costs. We have retained this distinction in the interpretation of the HHD estimates.

Additional Editor Comment 3 — Tariff payment units in Table 5

Comment: HHD BPT values are weekly, whereas in-centre HD and PD BPT values are per session and per day, respectively. The headings or footnotes should make these units explicit.

Response: Thank you. Table 5 now distinguishes “Estimated unit cost” from “BPT (payment unit)” and explicitly displays £/session, £/day or £/week in the relevant rows (Table 5, lines 310–322). The footnote explains that in-centre and satellite haemodialysis are benchmarked per session, peritoneal dialysis per treatment day, and HHD against a fixed weekly BPT irrespective of treatment frequency. Weekly values are derived using the stated treatment schedules, permitting comparison on a common weekly basis. We have also clarified in the Results (lines 326–337) that the HHD benchmark margin declines as frequency increases because its BPT is fixed per week. The figures are described as benchmark margins rather than actual incremental reimbursement.

Additional Editor Comment 4 — Discussion references

Comment: Several Discussion citations do not match the studies described, notably the Value in Health analysis, international HHD comparisons and broader CKD economic evaluations. Please verify and correct citation numbering.

Response: We agree and have systematically corrected the citations and reference numbering. The UK integrated home dialysis cost-effectiveness analysis by Erbe and colleagues is now cited as reference 10 (Discussion, lines 352–355); the French comparative HHD study by Rostoker and colleagues as reference 11 (lines 355–357); and the CKD economic burden review by Jha and colleagues as reference 12 (lines 358–361). The subsequent references have been renumbered consistently, the duplicated UK Kidney Association utilities entry has been removed, and the reference list now contains 20 entries. The Welsh costing studies remain correctly cited as references 15 and 16 (lines 385–394).

Journal requirement — Reference list review

We have reviewed the reference list for completeness, duplication and correspondence with in-text citations, and have corrected the numbering and removed the duplicate reference.

We thank you again for your constructive comments and hope that these clarifications resolve the remaining concerns.

Attachment Submitted filename: PLOS_ONE_Response_to_Reviewers.docx

# Editor report: Decision Letter 2

**DOI:** 10.1371/journal.pone.0362592.r005

**Authors:** Wisit Kaewput

27 Oct 2026

The true cost of dialysis modalities: a detailed analysis from a UK renal centre

PONE-D-26-32349R2

Dear Dr. Kolhe,

We’re pleased to inform you that your manuscript has been judged scientifically suitable for publication and will be formally accepted for publication once it meets all outstanding technical requirements.

Within one week, you’ll receive an e-mail detailing the required amendments. When these have been addressed, you’ll receive a formal acceptance letter and your manuscript will be scheduled for publication.

An invoice will be generated when your article is formally accepted. Please note, if your institution has a publishing partnership with PLOS and your article meets the relevant criteria, all or part of your publication costs will be covered. Please make sure your user information is up-to-date by logging into Editorial Manager at Editorial Manager® and clicking the ‘Update My Information' link at the top of the page. For questions related to billing, please contact billing support.

If your institution or institutions have a press office, please notify them about your upcoming paper to help maximize its impact. If they’ll be preparing press materials, please inform our press team as soon as possible -- no later than 48 hours after receiving the formal acceptance. Your manuscript will remain under strict press embargo until 2 pm Eastern Time on the date of publication. For more information, please contact onepress@plos.org.

Kind regards,

Wisit Kaewput, MD, FRCP, FRCPSG, FASN, FAcadMEd

Academic Editor

PLOS One

Additional Editor Comments (optional):

Thank you for your careful revision and detailed responses to the editorial comments.

The four outstanding concerns have been satisfactorily addressed. The revisions have improved the numerical consistency, methodological transparency, and clarity of the manuscript.

Reviewers' comments:

# Editor report: Acceptance letter

**DOI:** 10.1371/journal.pone.0362592.r006

**Authors:** Wisit Kaewput

PONE-D-26-32349R2

PLOS One

Dear Dr. Kolhe,

I'm pleased to inform you that your manuscript has been deemed suitable for publication in PLOS One. Congratulations! Your manuscript is now being handed over to our production team.

At this stage, our production department will prepare your paper for publication. This includes ensuring the following:

* All references, tables, and figures are properly cited

* All relevant supporting information is included in the manuscript submission,

* There are no issues that prevent the paper from being properly typeset

You will receive further instructions from the production team, including instructions on how to review your proof when it is ready. Please keep in mind that we are working through a large volume of accepted articles, so please give us a few days to review your paper and let you know the next and final steps.

Lastly, if your institution or institutions have a press office, please let them know about your upcoming paper now to help maximize its impact. If they'll be preparing press materials, please inform our press team within the next 48 hours. Your manuscript will remain under strict press embargo until 2 pm Eastern Time on the date of publication. For more information, please contact onepress@plos.org.

You will receive an invoice from PLOS for your publication fee after your manuscript has reached the completed accept phase. If you receive an email requesting payment before acceptance or for any other service, this may be a phishing scheme. Learn how to identify phishing emails and protect your accounts at https://explore.plos.org/phishing.

If we can help with anything else, please email us at customercare@plos.org.

Thank you for submitting your work to PLOS One and supporting open access.

Kind regards,

PLOS One Editorial Office Staff

on behalf of

Dr. Wisit Kaewput

Academic Editor

PLOS One
